# From Network Sensors to Intelligent Systems: A Decade-Long Review of Swarm Robotics Technologies

**DOI:** 10.3390/s25196115

**Published:** 2025-10-03

**Authors:** Fouad Chaouki Refis, Nassim Ahmed Mahammedi, Chaker Abdelaziz Kerrache, Sahraoui Dhelim

**Affiliations:** 1 Department of Computer Science, University of Batna 2, Batna 05078, Algeria; fouad.refis@etu.univ-batna2.dz; 2 The Interdisciplinary Centre for Security, Reliability, and Trust (SnT), University of Luxembourg, L-1855 Luxembourg City, Luxembourg; nassim.mahammedi@uni.lu; 3 Laboratoire d’Informatique et de Mathématiques, University Amar Telidji of Laghouat, Laghouat 03000, Algeria; 4 The School of Computing, Dublin City University, D09 V209 Dublin, Ireland

**Keywords:** swarm robotics, systematic review, swarm intelligence, metaheuristics, robot operating system (ROS)

## Abstract

Swarm Robotics (SR) is a relatively new field, inspired by the collective intelligence of social insects. It involves using local rules to control and coordinate large groups (swarms) of relatively simple physical robots. Important tasks that robot swarms can handle include demining, search, rescue, and cleaning up toxic spills. Over the past decade, the research effort in the field of Swarm Robotics has intensified significantly in terms of hardware, software, and systems integrated developments, yet significant challenges remain, particularly regarding standardization, scalability, and cost-effective deployment. To contextualize the state of Swarm Robotics technologies, this paper provides a systematic literature review (SLR) of Swarm Robotic technologies published from 2014 to 2024, with an emphasis on how hardware and software subsystems have co-evolved. This work provides an overview of 40 studies in peer-reviewed journals along with a well-defined and replicable systematic review protocol. The protocol describes criteria for including and excluding studies and outlines a data extraction approach. We explored trends in sensor hardware, actuation methods, communication devices, and energy systems, as well as an examination of software platforms to produce swarm behavior, covering meta-heuristic algorithms and generic middleware platforms such as ROS. Our results demonstrate how dependent hardware and software are to achieve Swarm Intelligence, the lack of uniform standards for their design, and the pragmatic limits which hinder scalability and deployment. We conclude by noting ongoing challenges and proposing future directions for developing interoperable, energy-efficient Swarm Robotics (SR) systems incorporating machine learning (ML).

## 1. Introduction

The world outside our minds is the primary source of knowledge for us as humans. Since the earliest stages of human history, nature has profoundly inspired and shaped our thoughts. It continues to be a source of innovation, influencing everything from the tools we use to the stories we tell. This occurs through cognitive processes that transform environmental observations into actionable knowledge. Artificial intelligence (AI) was inspired by the biological intelligence of humans, and Swarm Robotics (SR) was inspired by how nature’s swarms function. SR draws inspiration from social insects such as ants, bees, and termites, which coordinate and perform complex tasks through self-organization without centralized control [1,2,3,4,5]. Every technology relies on a foundation of hardware and software for effective implementation.

Similarly, SR technology is built upon hardware and software inspired by natural Swarm Intelligence (SI) algorithms. These so-called metaheuristic algorithms address various optimization problems. A metaheuristic is an “iterative generation process which guides a subordinate heuristic by combining intelligently different concepts for exploring and exploiting the search spaces using learning strategies to structure information to find efficiently near-optimal solutions” [6]. The term is derived from two Greek words, meta and heuristic, and the concept was coined by Glover in 1986 [7]. As already mentioned, metaheuristic algorithms are used to solve optimization problems, which are problems that “ask for minimal or maximal values of an objective function on a given domain” [8]. However, hardware poses several challenges. No standard architecture for SR exists due to the diversity of SR systems. Hardware refers to the physical elements or devices used to implement Swarm Robotic systems. These elements include sensors, actuators, processors, communication modules, energy sources, and mechanical structures. The goal of both hardware and software in SR is to build robots that can mimic the complex swarm behavior of social animals, so the robots need to exhibit swarm characteristics, which include robustness, flexibility, scalability, decentralization, and autonomy [2,9,10].

Despite the considerable advances in hardware and software design associated with SR systems, challenges persist. The lack of a unified framework for hardware components, software architectures, or communication protocols in SR systems makes it difficult to compare research findings, develop cost-effective solutions, and transition SR systems from research labs to real-world applications. Furthermore, this lack of standardization limits cross-platform applications. There have been literature reviews providing insight into the different aspects of SR. However, few have offered a holistic review that considers both hardware and software in SR systems, while also accounting for the economic and practical constraints of developing SR systems.

**Figure 1:**
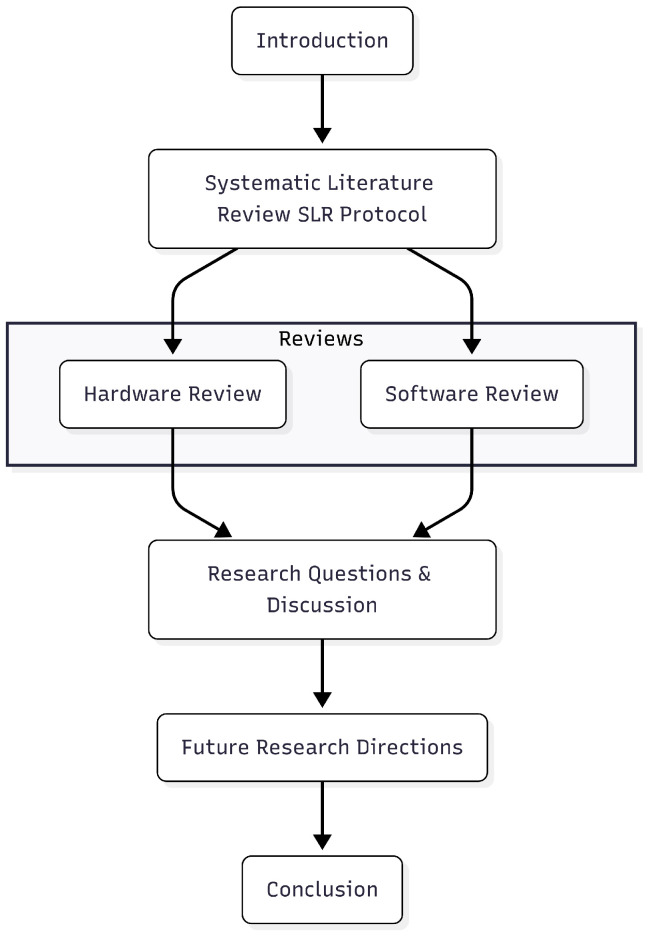
Paper structure diagram.

In this paper, we conduct a systematic review to introduce the state-of-the-art for both the hardware and software elements of SR systems, focusing on the past decade of research. We chose a systematic review because it is a transparent and structured method to compile and evaluate research findings on a specific topic or question. The aim is to minimize the bias associated with single studies and non-traditional reviews [11]. A systematic review “renders itself amenable for replication” [12]. In contrast to previous reviews that treat hardware and software separately, our review illustrates the interplay between the two. We compare different hardware designs and software systems that embody Swarm Intelligence, such as the Robot Operating System (ROS), and then we note major trends, challenges, and potential directions for standardized and optimized SR systems for future applications. As illustrated in Figure 1, the rest of this article is organized as follows: in Section 2 we present the SLR (systematic literature review) protocol, including research questions, exclusion and inclusion criteria, and how we performed the protocol; in Section 3, we then conduct a hardware type review of SR, differentiating the hardware components of SR (sensors, actuators, communication acquisition modules, and power source) which differed across SR types. Section 4 explores the software used in the field of SR. Section 5 answers the research questions and synthesizes the key trends and challenges in the SR research field, focusing on standardization and cost-effective solutions. It also explores future directions, including machine learning integration and energy-efficient designs. Section 6 explores future research directions and, finally, Section 7 provides the conclusion.

## 2. The SLR Protocol

A comprehensive and rigorous systematic literature review starts with a detailed and predefined protocol that outlines all the steps and methods to be followed during the review. This protocol includes research questions, exclusion and inclusion criteria, and protocol execution procedures.

### 2.1. Research Questions

- What are the key standardization barriers that prevent Swarm Robotics deployment in real world applications?
- How do hardware/software integration choices affect swarm performance and scalability?
- Which design patterns show up in Swarm Robotics implementations that are successful versus those that are unsuccessful?

### 2.2. Exclusion and Inclusion Criteria

- The papers must be published between 2014 and 2024.
- The papers are written in English.
- The papers are the results of searching in Google Scholar, Google search engine, and Semantic Scholar.
- Exclude duplicate papers that contain redundant information.
- Papers must be four pages or longer to exclude works lacking scientific methodology and sufficient detail.
- Papers that are inaccessible are excluded.
- Papers that do not contribute to the research after reviewing them are excluded.

### 2.3. Quality Assessment Criteria

1. Research Design and Methodology:
  - Clearly defined research objectives: Does the paper outline its contributions and research goals?
  - Description of methodology: Is the development or experimental methodology sufficiently described?
  - Software and hardware validation: Have the suggested software and hardware components undergone adequate testing and validation?
2. Technical Contribution:
  - Innovation and novelty: Does the paper offer new software or hardware solutions or enhancements?
  - Technical depth: Is the technical content rigorous and detailed enough?
3. Experimental Evaluation:
  - Experiments: Are they appropriate and well-designed?
4. Relevance and Impact:
  - Swarm Robotics relevance: To what extent does the work relate to the field of Swarm Robotics?
  - Can the proposed solution be practically implemented?

### 2.4. Execution

**Figure 2:**
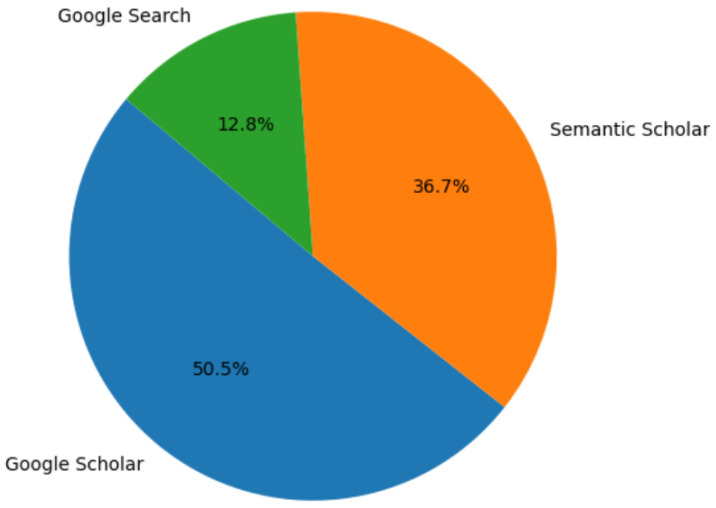
Distribution of the 196 scientific papers across different search engines.

**Figure 3:**
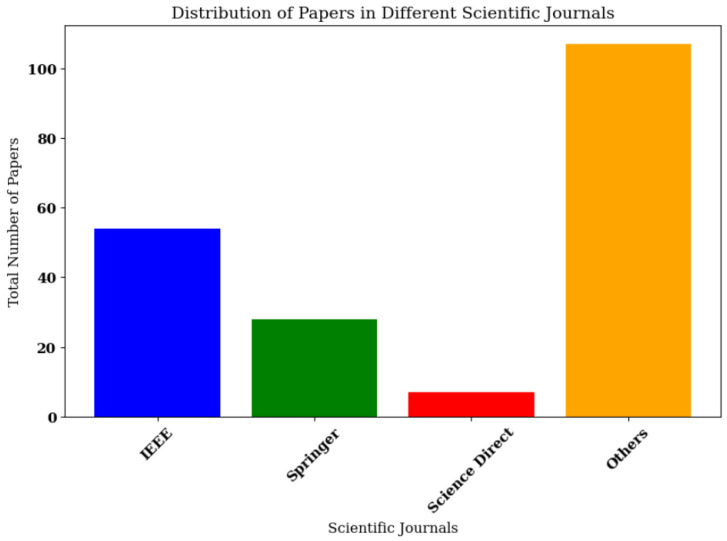
Distribution of the scientific papers regarding the scientific journals.

**Figure 4:**
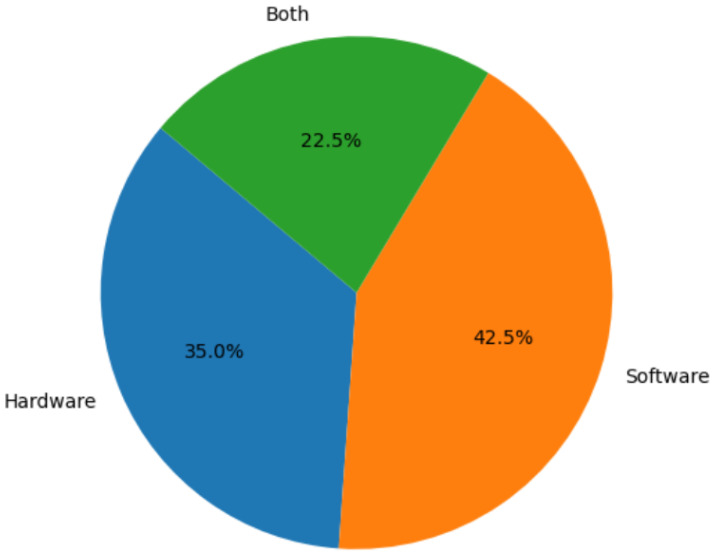
Distribution of the 40 selected papers regarding their focus area (14 hardware, 17 software, 9 both hardware and software.

Keyword selection was based on the study subject and applied to paper titles. In Google Scholar and Semantic Scholar, the following keywords were used: (swarm and (robot or robots or robotics) and (design or hardware or software)) + how to create an SR. In Google search engine, the following was used: hardware design implementation of Swarm Robots. Figure 2, Figure 3 and Figure 4 illustrate the entire process, including the distribution of the articles with respect to the search engines, the scientific journals, and the focus area.

The results were:

99 papers –> Google Scholar.

72 papers –> Semantic scholar.

25 papers –> Google search engine.

196 paper –> total.

**Figure 5:**
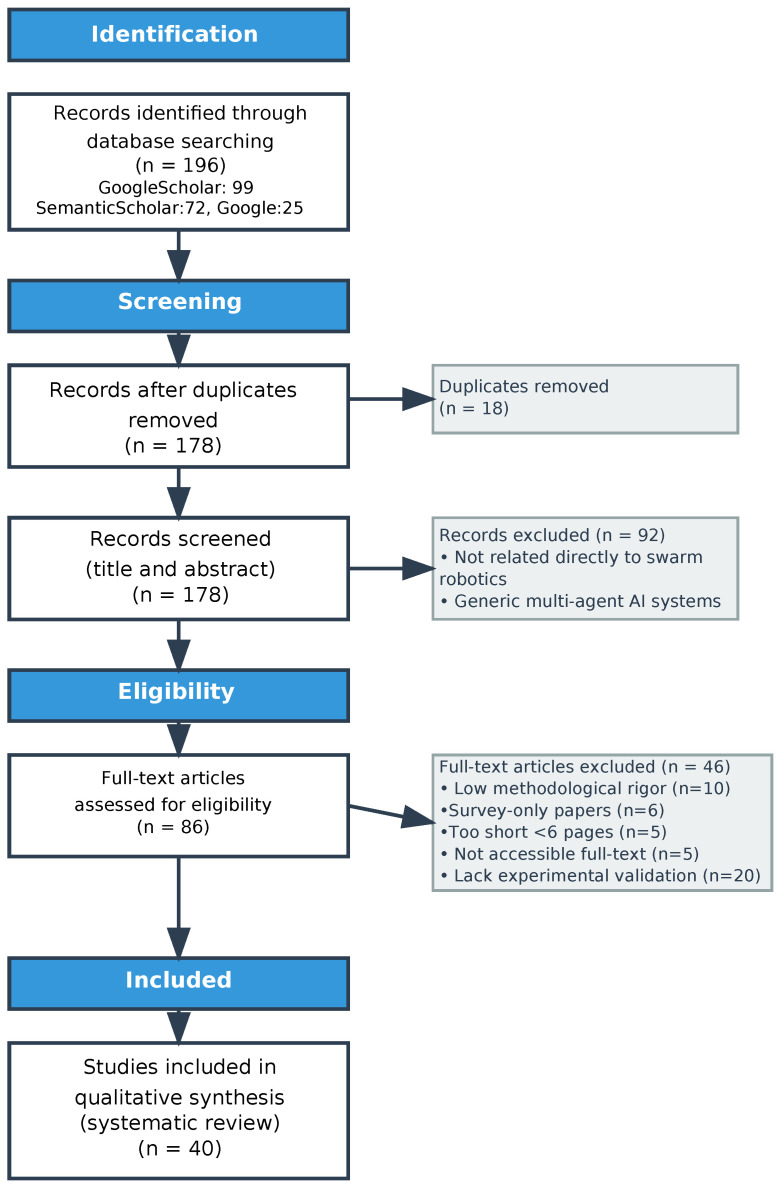
Execution diagram (PRISMA-style, see Supplementary Materials).

Following PRISMA style [13] and after applying the criteria outlined in Section 2.2 and Section 2.3, 40 out of 196 papers met the requirements, representing 20.41% of the total (see Figure 5).

Before proceeding, it is worth noting that the SR field needs an “urgent standardization in several aspects, including the robots hardware and software” as Nedjah and Junior stated [14]. According to them, this lack of standardization is one of the main reasons SR applications are not yet in our daily lives. The goal is to design a swarm of robots with low-cost and that are easy to control as to yield intelligent collective behaviors; there are attempts to solve the problems that are facing the standardization but they are ad hoc. Each SR model has its own sensors, programming language, and actuators. This diversity complicates project migration across platforms due to hardware or software incompatibilities. The goal of unification has yet to be accomplished. However, there are some promising attempts like Villemure et al.’s [15] hardware/software open platform for SR development. The process of building SR is divided into two parts: the hardware design and the software design.

## 3. Hardware Design

**Figure 6:**
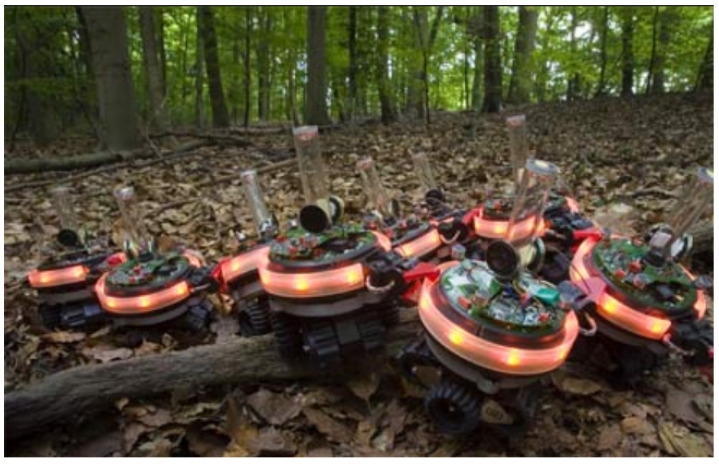
A swarm of robots [18].

While the software is the intelligent core, providing a virtual environment that governs the robot’s operations, it is the hardware that executes the instructions dictated by the software, translating them into physical actions. The challenge with hardware is the significant variation required for different environments: in the air (aerial SR), on land (terrestrial SR), or underwater (aquatic SR). An example of terrestrial SR is illustrated in Figure 6. The hardware variations in Swarm Robots are also driven by the specific requirements of the task, as demonstrated by the experimental results of Salman et al. [16]. For example, cameras and vision sensors are more compatible with navigation tasks, while infrared or thermal sensors are better suited for rescue operations, and chemical sensors are useful for environmental monitoring. Furthermore, building SR systems under economic constraints differs significantly from building them without such constraints, as hardware prices vary, and the hardware differs between homogeneous and heterogeneous SR systems [17]. As noted by Salman et al. [16], the hardware specifications of each individual robot, the design of the control software orchestrating their behavior, and the optimal swarm size are heavily contingent upon the distinctive nature of the collective mission at hand, as well as the economic constraints imposed on the project. While “a universal swarm design methodology does not exist” [16], a general hardware architecture is common across most swarm robots.

### 3.1. Sensors

**Figure 7:**
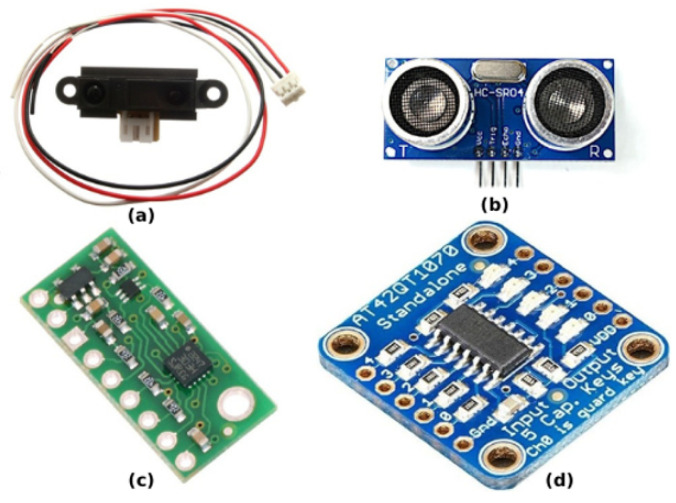
Sensors used in SR. (**a**) Sharp GP2Y0A21YK IR proximity sens [19]. (**b**) HC-SR04 Ultrasonic Distance Senso [20]. (**c**) LSM303D 3D Compass and Accelerometer Carrier with Voltage Regulato [21]. (**d**) Adafruit Standalone 5-Pad Capacitive Touch Sensor Breakout—AT42QT1070 [22].

The sensory unit used by the SR is essential for perceiving “the surrounding environment to the controller—a process known as mapping” [17]. This process is vital for accomplishing tasks such as detecting and avoiding obstacles, detecting neighboring robots, and navigation. It is also used to collect sensory data that allows the SR to make decisions. Each SR system can be equipped with various sensors (see Figure 7), such as cameras, proximity sensors, range sensors, GPS, humidity sensors, temperature sensors, and chemical sensors. (sensor categories, their names, the robots equipped with them, power consumption, and sensor prices are listed in Table 1).

Variations in robotic sensors may result in robots perceiving information differently, which influences the control unit to generate different actuation commands for the actuators based on the sensory input. The infrared (IR) proximity sensor is the most commonly used sensor, as it is small, easy to mount, and can detect objects at short ranges of 5–15 cm, depending on the object’s color [30]. Nedjah and Junior note that IR sensors are present in most robots [14]. These sensors work by emitting infrared light, which is reflected off objects—closer objects cause stronger reflection intensity. (The mathematics behind the work of IR sensors have been illustrated in Equation (1), which represents the sensor response function, which is a response to an IR sensor. Equation (2) represents the cumulative sensor response and Equation (3) represents the sensor model in Colias SR).

Reviewing SR hardware papers from 2014 to 2024, we observe different sensors implemented in SR projects. In the cellular project, the robots were equipped with a downward-facing camera [30]. The UBswarm, developed at the RISC lab at the University of Bridgeport, uses ultrasonic as well as photoelectric (infrared) proximity sensors [24]. In the Zooids project, a flexible electrode is wrapped and embedded within the 3D-printed housing, facilitating capacitive touch sensing. An integrated capacitive touch sensing circuit (Atmel AT42QT1070) is included to detect user touch [26].

Aquatic SR systems, as described by Costa et al. [25], incorporate sensors into every robot, including temperature sensor, digital compass, and a GPS receiver. The GPS receiver, a GlobalTop FGPMMOPA6H module, provides position updates with 5 Hz frequency and interfaces with the single-board computer via UART protocol. It is equipped with a 26 dB gain GPS antenna for more powerful signal quality and position accuracy of ±3 m. The digital compass unit, an STMicroelectronics LSM303D magnetometer, provides information by compensating based on the robot’s pose. Due to interference, the magnetometer is placed in a secondary enclosure in the vessel’s prow. Temperature data are obtained from the onboard SBC temperature sensor and a Maxim DS18B20 sensor, positioned in the vessel’s bottom, to measure water temperature. The DS18B20 sensor provides digital temperature readings with 12-bit resolution and interfaces with the SBC via the One-Wire standard protocol.

In Bartmess et al.’s [33] project to build fast, low-cost swarm robots, a webcam is positioned above a table to observe the tabletop surface and the robots on it. The webcam connects to the computer through a USB interface and is an off-the-shelf component that adheres to the cost constraints set for the vision system. The webcam derives its power supply from the USB connection. In Shang’s PhD thesis [34], the robot is equipped with two infrared photoelectric sensors. The responses of each individual reflective point within the sensor’s viewing range are added up to determine the IR sensor’s output magnitude:

(1) $$S\left(x,\theta \right)=\frac{\alpha }{x^{2}}\cos\left(\theta \right)$$

where S(x) is the IR sensor’s response to an individual reflective point.

θ is the incidence angle of the reflected light.
*x* is the distance from the reflective point.
*v* is the IR sensor’s viewing angle.
α is the amplifier’s gain and determines the sensor’s sensitivity.

(2) $$V_{\mathrm{IR}}=\sum_{n=1}^{n}S\left(x_{n},\theta_{n}\right)+\beta$$

*n* is the quantity of dots within the viewing angle of the infrared sensor.
β simulates the impact of ambient light and the offset of the sensor’s output.

In the Colias SR [27], only IR proximity sensors are used. The following formula serves as a mathematical model for the reflected infrared value that a sensor measures:

(3) $$s\left(x,\theta \right)=\frac{\alpha_{c}\cos\theta }{x^{2}}+\beta_{c}$$

s(x,θ) is the sensor output signal.
*x* is the distance of the obstacle.
θ is the angle of incidence with the surface.
$\alpha_{c}$ includes several parameters, such as the reflectivity coefficient, output power of emitted IR, and sensor sensitivity.
$\beta_{c}$ is the amplifier’s offset value plus the effect of ambient light.

Another aquatic SR project, “Jeff,” by Mintchev et al. [29], involves an autonomous underwater vehicle (AUV) equipped with several sensors and communication systems. Underwater navigation in this system relies on a pressure sensor and a gyroscope to measure depth. An accelerometer and a magnetometer are used to control orientation and locomotion, and additional sensor payloads (e.g., temperature sensors, chemical concentration sensors, and cameras) can be integrated into Jeff’s modular shell. The SwarmUS project is equipped with an A2M8 RPLidar and a D400 series Realsense camera [15].

In their project, Mustafa et al. [35] utilize the HC-SR04 ultrasound sensor from ElecFreaks. Operating at 5V DC, the sensor uses sonar to measure distances to objects. Each module consists of an ultrasonic transmitter, receiver, and control circuit. The sensor emits ultrasound pulses at a constant frequency of 40 kHz with 8 cycles per burst and captures echoes lasting milliseconds. With an accuracy of about 3 mm and a pulse travel range of 2–500 cm, the sensor is used for collision avoidance with both static and dynamic objects, including other robots in the swarm and the physical boundaries of the 3D environment.

The 25 g pico quadrotor with a protective cage is equipped with a wireless camera installed on the bottom, capable of providing real-time video feedback [36]. Another SR project has been equipped with additional introspective sensors (specifically motor current and temperature sensors) [31]. The S-Bot robot uses a variety of sensors for different purposes, including the Sharp distance sensor 2Y0A21, the ultrasonic distance sensor HCSR04, and a general-purpose proximity sensor that uses an infrared emitter and receiver pair. The Sharp GP2Y0A21YK0F measures distances between 10 cm and 80 cm, with its output voltage matching the detection distance. In contrast, the HCSR04 has a range of 2 cm to 400 cm with an accuracy of 3 mm. Through the use of an onboard potentiometer, the general-purpose proximity sensor’s operating range can be adjusted from 2 cm to 15 cm. Noted for its compact design and low power consumption [23,37], the Bulubot prototypes also utilize two types of sensors. Firstly, the Sharp GP2Y0A41SK sensor, in order to detect obstacles, a short-range infrared proximity sensor is employed. Second, for light following, a 10 mm light-dependent resistor (LDR) is used [38]. Bump sensors, mounted upward, allow the direction of impact to be detected on the bottom circuit board, with an average error of 8.1°. These bump sensors are used for obstacle avoidance and to estimate the angle of impact [39]. The mROBerTO platform features both a proximity-sensing module and a swarm-sensing module [40]. Their research explored SR systems for material handling tasks and enhancing solar energy conservation efforts. In Kumar et al.’s work [41], the SR is equipped with IR proximity sensors, temperature sensors, humidity sensors, and LDRs.

The RiBot project places an IR sensor (TSOP75436WTT) on the back of the robot [42]. The HeRo robot [28] avoids obstacles and collisions with other robots by using only infrared proximity sensors. The three TCRT5000 long-range sensors that make up the IR sensory system are positioned in front of the robot and have a 20 cm range. In the simulation conducted by Shang et al. [43], two downward-pointing IR sensors are located at the front of the robot. For another project [32], Infrared sensors and switches were combined. An Analog to Digital Converter (ADC) module on the ATtiny85 microcontroller detects and measures voltage variations across specific I/O pins. This capability is used to determine the robot’s distance and read the voltage from an infrared receiver.

### 3.2. Actuators and Locomotion Mechanisms

**Figure 8:**
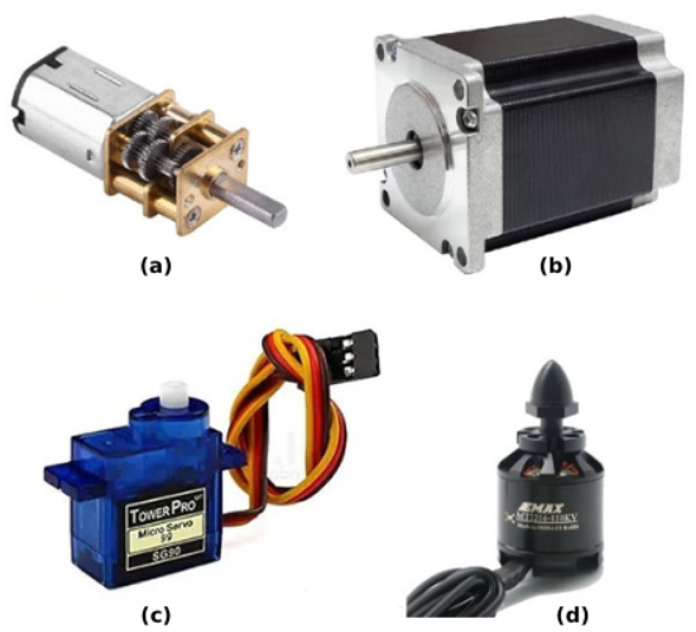
Locomotion mechanisms used in SR: (**a**) DC 6V Gear Motor High Torque [45]. (**b**) Stepper motor [46]. (**c**) TowerPro SG 90 Micro Servo Motor [47]. (**d**) EMAX Multicopter motor MT2213 [48].

Like muscles which enable movement and coordination in humans, actuators enable movement and control of the robot’s joints and linkages (see Figure 8) via control signals that it receives from the controller. Actuators help the robot to resist gravity, inertia, and other external forces when in operation [44]. In Mustafa et al.’s work [35], wheels and servomotors are the two primary parts of their SR platform. Two side wheels that are driven by servomotors that rotate continuously provide the robot with movement. Servomotors, in contrast to conventional motors, can be individually controlled and only need the rotational angle to move. An omnidirectional ball caster wheel supports the robot’s rotation, enabling it to swivel in any direction.

The SwarmUS platform [15] focuses on the coordination, communication, and localization elements required for swarm behavior rather than directly addressing actuators. Wheels are the Pioneer 2DX with SwarmUS’s primary feature. Conversely, “Jeff,” the aquatic SR [29], is adaptable for underwater swarm operations because it uses a combination of DC motors and specially made mechanisms to control movement. One DC motor regulates the cam and piston mechanism in Jeff’s buoyancy system, allowing for depth control and up/down movement. To promote attraction and repulsion in the docking processes, the docking system also employs a DC motor which controls the orientation of a magnet inside the docking station.

Other aquatic SR actuators are represented in the work of Costa et al. [25], where two DC motors each drive a propeller through a shaft. Two particular models are mentioned in the paper: the Emax 2215/25 950 kv 2-3S and the NTM Prop Drive Series 28–30 A 750 kv/140 W. Furthermore, the HobbyKing 50 A Boat ESC 4 A UBEC and other electronic speed controllers (ESCs) regulate the direction and speed of the DC motors, enabling accurate robot movement.The Colias robot [27] is driven by two tiny DC motors that use direct gears and two 2.2 cm-diameter wheels, allowing it to reach a top speed of 35 cm/s. Using distinct H-bridge controllers and pulse-width modulation (PWM) techniques, each motor’s rotational velocity is individually controlled.

DC motor drivers power each motor, with an average power consumption of 35 ± 5 mA under no-load conditions and up to 150 ± 20 mA when stalled. In their work on building fast, low-cost SR, Bartmess et al. [33] used two DC motors per robot for propelling the robot around a table. These motors are connected to the wheels, allowing differential drive by varying the speed of each wheel. The design includes motor controller ICs that connect to the DC motors. These ICs receive PWM signals from the SoC and change them into signals that can drive the motors, which lets you control speed and direction.

In the Zooids project [26], the actuators are micro DC motors with wheels, which allow the robots to move and navigate on flat surfaces, supporting dynamic and interactive swarm user interfaces. The UB Robot Swarm [24] uses DC motors for driving the robot’s wheels, with various types of motors such as Solarbotics gear motors, micro-metal gear motors, and Tamiya gearbox motors. For manipulating things with a robot arm, geared DC motors are also used. Hitec HS-422 servo motors move the arm and gripper, giving the robots precise control over movement and enabling them to grasp objects.

The Cellulo robots [30] are equipped with omnidirectional ball drive actuators for locomotion, enabling holonomic movement, meaning they can move in any direction and change direction instantaneously. Patil et al. [17] reviewed actuators used in various swarm robot platforms and concluded that DC motors are widely used for driving wheels and tracks on platforms like E-puck, Alice, Sumobot, Swarm-Bot, AutoBot, and CYBOTS. Some platforms, like E-puck and Nanokhod, use stepper motors for precise control. Servo motors, on the other hand, allow for positional and motion control for locomotion and other tasks. Microrobots use piezoelectric actuators because they are small and accurate. Robots like TerminatorBot use gear motors to move things more powerfully. In his SR design, Shang [34] used DC motors and two wheels. The pico quadrotor [36] uses four DC brushed motors as its main actuators in aerial SR. These motors create the thrust and torque needed to control movement and orientation. The Robotarium’s GRITSbots [31] use stepper motors as their main actuators. This lets them move with great accuracy and run a variety of swarm robotics algorithms correctly. The S-Bot robot [23] has a caster wheel on the front for support and two DC geared motors to control its movement. The drive motors let the robot turn left or right and move forward and backward. Distance sensors help the motor system work better. In his master’s thesis, Demir [38] used four DC motors, each operating at 60 rpm, alongside two TB6612FNG motor drivers to drive the robot’s legs.

The r-one robot [39] uses DC gear motors to move the wheels of the robot, providing motion and manipulation. The motors have a 100:1 gearbox with them for precise speed control and torque. The robot’s gripper attachment uses an S-75 sub-micro servo motor to control the paddles of the gripper to allow for grasping and releasing objects with varying force.

In mROBerTO [40], two 4 mm nano coreless DC motors are used in a differential drive configuration, eliminating the need for additional gearing, simplifying the design and reducing size. Vibration motors, though not used in mROBerTO, are mentioned as an alternative locomotion method used in Kilobot. However, Although vibration motors are not used in mROBerTO, they are employed in Kilobot as an alternative locomotion method. However, they offer lower precision and are less effective for long-distance movement compared to DC motor-based systems.

Kumar et al. [41] worked on SR for solar energy conservation, where they employed DC motors to drive the robots’ wheels, enabling movement. They also used L293D motor driver ICs to control the speed and direction of the DC motors. RiBot [42] is equipped with a micro step gear motor (“MF03G” by Seiko Precision Inc.) for actuating the robot’s tail (caudal peduncle), allowing it to mimic the tail movements of a real zebrafish. HeRo [28] uses two SG90 micro servo motors modified for continuous rotation to drive its wheels and control its movement.

The final SR project introduced is by Abuelhaija et al. [32], who used two DC motors per robot. These motors facilitate movement and they are controlled by a DRV8833 dual H-bridge motor driver chip. This setup allows for bidirectional control of the motors, enabling the robots to move forward, backward, and turn.

### 3.3. Communication and Networking

**Figure 9:**
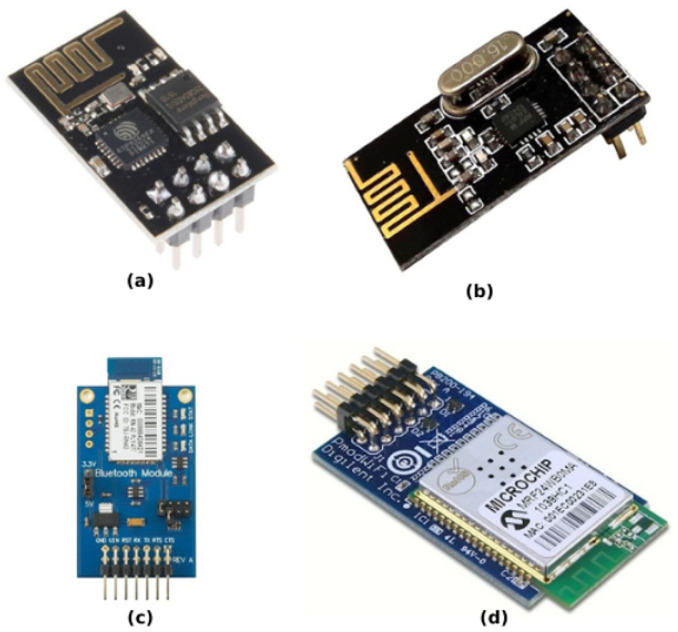
Communication and networking hardware used in SR. (**a**) ESP8266 WiFi Modul [49]. (**b**) 2.4 GHz Wireless Transceiver nRF 24L0 [50]. (**c**) RN-42 Bluetooth Module [51]. (**d**) Pmod WiFi: WiFi Interface 802.11 g [52].

“In multi-robot systems, communication media are used for inter-robot communication to share information and make collective decisions” [23]. Most often, wireless communication (see Figure 9) is employed when a scenario involving mobile robots is completed [27]. The choice of communication module depends on several factors [24], including coverage, price, and power consumption.

- Communication Distance Capability: The X-Bee modules have more extensive communication range in comparison to the Bluetooth Bee modules.
- Data Transmission Speed: The PmodWiFi module, when mixed with the SPI interface, facilitates a higher rate of data transfer comparing to the X-Bee and Bluetooth Bee modules.
- Energy Consumption: Each module exhibits distinct power requirements.

Moustafa et al. [35] in their hardware robotic platform for very short distances (10 m or less) use an integrated communication interface for Bluetooth 4.1, and for larger areas (100 m), they use an 802.11n wireless LAN, and an FM receiver operating in the 65–108 MHz FM bands. These wireless capabilities are enabled by the Cypress CYW43438 wireless chip. The various wireless communication options are utilized for facilitating short- and long-range communication for the swarm robot system. SwarmUS, equipped with a communication system, utilizes a Wi-Fi network for data exchange, with broadcast messages for updating shared information and unicast messages for sending commands. It employs a messaging system based on Protobuf and RPC mechanisms to route communications between agents, hosts, and other system components [15]. Moving to the aquatic SR, Jeff [29] is equipped with blue LED units used for communication and distance sensing; each unit has two pairs of LEDs, one pair with 1m range and a 60° beam for communication, and the other pair with 0.5 m range and a 120° beam for obstacle detection. Inspired by the electrosensory capabilities of fish, fourteen electrodes enable communication and localization through electric field detection/interpretation, with a range of 250–500 mm; it also has two loudspeakers, one on each side, with a range of 0.5–1 m, and finally a microphone that receives acoustic signals from a floating station used for a virtual fence system to confine the swarm within a designated area. Colias [27] uses infrared (IR) technology for both communication and sensing, utilizing short-range IR bump sensors for basic obstacle avoidance, while the long-range IR proximity sensors provide both environmental sensing capabilities (obstacle detection and range estimation) as well as a means for direct communication between robots in the swarm. Another aquatic SR developed by Costa et al. [25] is equipped with a TP-Link TL-WN722N Wi-Fi adapter with a high-gain antenna, which allows robots to communicate wirelessly with each other over a range of 40 m on the water. The fast, low-cost 16 SR developed by Bartmess et al. [33] is equipped with a Wi-Fi router capable of transmitting a 2.4 GHz signal to the robots. The router communicates with the robots using a UDP connection, and the system enables rapid and frequent communication. It establishes a connection with the computer through an Ethernet cable, facilitating simultaneous connections with up to 16 devices. Remarkably, it can transmit 16 packets, each with a size of 16 kilobytes, within a mere 100 ms. These capabilities are attributed to the Zooids component. Zooids SR by M. Le Goc et al. [26] are equipped with two main elements of communication and networking hardware: 2.4 GHz Radio Chip (Nordic nRF24L01+), which enables wireless centralized communication between each Zooid, and a master computer. This also allows the information status to be sent back the master. The UB Robot Swarm [24] uses several communication modules depending on the specific robot and its role within the swarm. X-Bee modules: They offer wireless communication for both indoor and outdoor environments. They operate using serial communication (Tx/Rx) which are compatible with other communication modules.

- Bluetooth Bee Modules: Like X-Bee, they use serial communication for short-range communication within the swarm.
- PmodWiFi modules: Provide wireless communication through WiFi connectivity. They use the SPI mode to transmit data and receive it, offering faster data rates compared to serial communication.

The Cellulo SR project [30] uses wireless RN-42 Bluetooth for communication. Patil et al. in their review on SR hardware [17] mention various communication and networking options used in swarm robotics, each with its own advantages and drawbacks. “Short-Range Communication”, like infrared (IR) transceivers/sensors, is low-cost, has simple implementation, and is excellent for short-range robot-to-robot communication and obstacle detection, but its range is limited, susceptible to interference from ambient light, and requires line-of-sight. Also, ultrasonic sensors, which have a longer range than IR, can measure distance and angle, but are sensitive to object material and surface properties, and are less accurate than IR. For long-range communication, another hardware mentioned in the review is the Radio Frequency (RF) modules, which have longer range than IR or ultrasonic, and are good for complex environments, but have the potential to receive interference from other RF devices and they can be more expensive. Another one is Bluetooth: this can be relatively low-cost, have readily available modules, and is good for moderate data transfer, but it has a shorter range then the (RF). Finally, Wireless LAN (Wi-Fi), which has a high bandwidth and is good for large data transfers, but it has higher power consumption, potential interference issues, and is more expensive. In his thesis about hardware variation in Robotic Swarms [34], Shang categorizes the communication methods into three main types and provides examples of each technology category: (interaction via communication, interaction via sensing, and interaction via the environment).

The 25 g swarm pico quadrotors [36] primarily use ZigBee communication modules for wireless communication. The Robotarium [31] primarily relies on WiFi (IEEE 802.11 B/G/N) for communication between the robots and the central server. Each GRITSBot is equipped with an ESP8266 chip that provides WiFi capabilities with a bandwidth of up to 54 MBit/s. In the S-bot [31], as demonstrated, the CC2500 Serial Communication Module is a low-cost 2.4 GHz transceiver intended for extremely low-power wireless applications. The module is intended to operate in the ISM (Industrial, Scientific, and Medical) and SRD (Short Range Device) frequency bands, which are 2400–2483.5 MHz. The CC2500 features a standard configuration baud rate of 9600, a variable baud rate RS232 UART interface, and a programmable device address (255 per channel). In Demir’s master’s thesis [38] the focus of his work is on the mechanical design, leg optimization, and individual robot control for achieving flocking behavior, so he does not mention any communication hardware. In the r-one SR [39], eight infrared transmitters, eight infrared receivers, a 2.4 GHz radio with a 2 Mbps data rate, and a USB port are used for communication. In order to communicate with the user, the robot has three pushbuttons, three distinct arrays, and a VLSI1053 audio chip with MIDI playback for user interaction, each consisting of five LEDs emitting light. Another SR is mROBerTO [40]: it is equipped with two types of communication hardware wireless RF “ANT™” for low-power mesh networking and “BLE” for higher bandwidth and an infrared multi-channel system for local robot-to-robot communication and relative positioning. This suite of hardware enables effective communication and networking and localization capabilities for the SR system. In their work, Kumar et al. [41] primarily focus on Bluetooth modules for communication between the swarm robots and potentially with a computer. Each robot is equipped with a Bluetooth module, utilizing serial communication mode (Tx and Rx) for data exchange, enabling decentralized communication among the robots. HeRo [28] is equipped with a NodeMCU v3 board which has a built-in ESP8266 microprocessor that provides WiFi communication capabilities. This allows the robot to connect to a network and communicate with other devices, including a central computer running the Robot Operating System (ROS). In Abuelhaija et al.’s [32] work, the communication of the SR in this project relies on an indirect form of communication through infrared (IR) sensors: by measuring the strength of the received IR signal, a robot can estimate the distance to other robots within its line-of-sight. This information is then used by the control algorithm to guide the robot’s movement and achieve swarming behavior. Finally, the lack of unified communication standards continues to be a significant barrier to interoperability among heterogeneous swarms. In practice, robots typically employ a range of protocols (Bluetooth, ZigBee, IR, RF, Wi-Fi, etc.), but many of these are not interoperable and hinder wide-scale coordination. To alleviate this limitation, the prospect of adaptive communication methods is promising, whereby robots can select or change protocols based on the context under which they are operating (e.g., time-critical tasks, repetitive tasks), available bandwidth, and available energy. For example, low-energy RF modules may be activated for normal local coordination, while Wi-Fi may be activated when the robots need high-bandwidth for data aggregation or vision sharing. Using a hybrid approach will greatly reduce interoperability bottlenecks.

**Figure 10:**
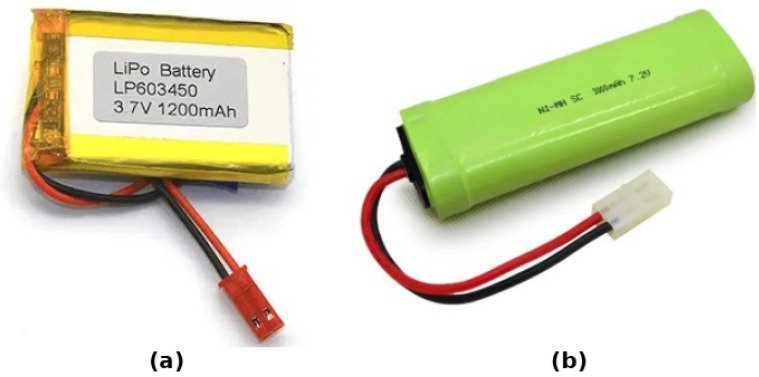
Power supply hardware used in SR. (**a**) 3.7 V-1200 mAh-lipo-battery [54]. (**b**) NiMH Rechargeable Battery (7.2 V–3000 mA) [55].

Power sources to a robot are like an engine to a car [53], so it is important to equip the SR with the appropriate power source (see Figure 10) depending on factors like the size of the robot and its mission. Moustafa et al. [35] used a rechargeable, power-efficient 3.7 V lithium-ion polymer battery in their SR platform. A boost converter is part of the system to raise the battery voltage to the necessary level, and a specialized lithium battery charger module that provides charging/discharging management, temperature control, and various protection features to ensure safe and reliable operation of the battery power source. SwarmUS [15] equipped with 11.1 V LiPo battery A DC/DC converter is used to step down the robot’s battery voltage to power the SwarmUS boards, and the Hiveboard distributes power to the connected Beeboards. Jeff the aquatic SR is equipped with eight Li-Po cells, each with an 880 mAh capacity. A lithium battery pack stores energy for up to 120 min of autonomy, located in Jeff’s stern. It also has battery status monitoring circuits, which are dedicated circuits that monitor the battery’s state of charge and remaining capacity. This information is crucial for Jeff’s cognitive capabilities, allowing it to make informed decisions about energy usage and potentially seek recharging when needed. Another SR is Colias [27], which utilizes A 3.7 V, 600 mAh lithium-polymer battery serving as the primary power source. This battery is expandable up to 1200 mAh for increased autonomy. The lower board of the robot houses a dedicated power management system. This system monitors and controls the power consumption of various robot functions. The aquatic SR developed by Costa et al. [25] utilizes two LiPo batteries, one dedicated to powering the motors and propulsion, and the other for control, processing, and sensing components. The control battery is regulated by an SBEC to provide a stable 5 V DC supply. The 16 fast, low-cost robots developed by Bartmess [33] use lithium-ion batteries (500 mAh); a circuit manages the charging process of the Li-Ion battery, preventing overcharging and undercharging to ensure safety and battery longevity. It also has a Voltage Regulator (LDL1117S33R): this component regulates the voltage from the battery to a steady 3.3 V, which is required for powering the ESP8285 SoC and other components. Each Zooids robot SR [26] is powered by a 100 mAh LiPo battery. The robots’ motors, radio module, microcontroller, and LED use the majority of their power. the Zooids are able to move for an hour and continue to function for even longer when used normally. The UB Robot Swarm [26] are powered by NiMH or LiPo batteries, chosen for their size, weight, and power characteristics. Power distribution and management involve considering the current consumption of individual components, as well as environmental factors and operational patterns. The Cellulo project [30] paper does not mentioned the details of the power supply of their SR, but it is a rechargeabale battery because Cellulo have a USB port for recharging it. In a review paper about SR hardware [17], the authors mention several power supplies like rechargeable lithium batteries, which are the most prevalent choice due to high energy density, compact size, and their light weight, especially lithium-polymer (Li-Po) batteries, which are favored for their safety and thin profile. SR typically operate on a voltage range of 5 V to 25 V DC power. They also mention some factors that influence battery choice:

- Robot size and weight: Smaller robots require smaller, lighter batteries.
- Power consumption: Robots with more sensors, actuators, and processing power need higher-capacity batteries.
- Mission duration: Longer missions necessitate batteries with longer run times.

### 3.4. Power Source

The energy supply hardware used in the pico quadrotor described in the paper [36] is a 3.7 V, 340 mAh Lithium Polymer (LiPo) battery. The robotarium (GRITSbots) [31] uses 400 mAh LiPo batteries: this is the onboard energy storage for each robot, allowing them to operate for up to 40 min, and a wireless charging system. S-Bot [23] has a 12-V rechargeable battery to power the whole system. Charging is performed using an external charger. The r-one robot [39] is equipped with the following energy supply hardware: a 3.7 V 2000 mAh lithium polymer (LiPo) battery, and the robot can be charged via the USB port or through a docking connector. The mROBerTO [40], utilizes the following energy supply hardware: three 3.7 V Li-Po batteries connected in parallel, a voltage divider, and an ADC port for battery monitoring. In their survey on Swarm Robotics material handling and how it can be used to conserve solar energy, Kumar et al. [41] pointed to Li-Po batteries as the primary power source, supplemented by miniature solar panels that harvest solar energy to recharge the batteries and extend their operational time. Additional hardware components like solar charge controllers and power meters are employed to manage and optimize energy usage and storage. The RiBot [42] uses a small 40 mAh rechargeable LiPo battery as its main power source. The battery can be conveniently recharged through contacts in the robot’s “eyes” without needing to disassemble the device. With continuous tail movement, the battery provides around 23 min of runtime, while intermittent tail usage extends the operating time beyond 1 hour. The HeRo SR is equipped with a 3.7 V 1000 mAh Li-Po battery and 5 V step-up boost converters; this converter efficiently steps up the voltage from the 3.7 V battery to the required 5 V level. Finally the paper from Abuelhaija et al. [32] describes their SR as being powered by two rechargeable 4.2 V lithium-ion batteries, with one battery dedicated to powering the motor circuit, while the other supplies energy to the control circuit. This separation is likely made to isolate potential electrical noise from the motors and ensure stable power delivery to the sensitive control electronics.

### 3.5. Summary of the Hardware Section

Due to their affordability and ease of integration, infrared proximity sensors remain the most widely used sensing technology. However, more recent swarm robotics platforms show a shift toward multi-modal sensing systems to support increasingly complex or dynamic mission needs. The most widely used actuator technology in terrestrial systems for locomotion is still linear DC motors with differential drive configuration. Advanced lightweight actuator technology and specialized propulsion mechanisms are features of both aquatic and aerial platforms. As the need for extremely agile platforms that can function in confined or unstructured environments grows, omnidirectional mobility is becoming a desired feature.

**Figure 11:**
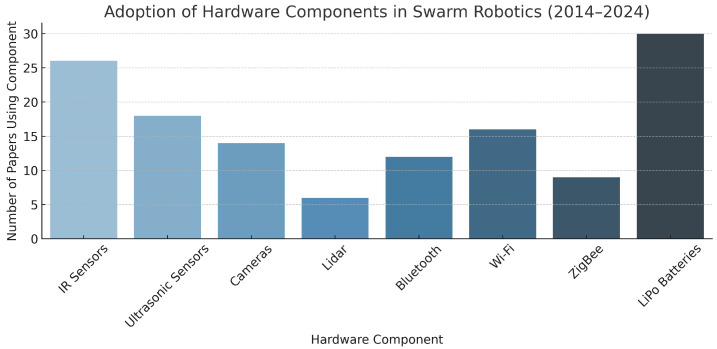
Summary of the adopted hardware components across the reviewed swarm robotics platforms between 2014 and 2024.

Communication technologies are very diverse and fragmented, with platforms utilizing various protocols as illustrated in Figure 11 (e.g., Wi-Fi, Bluetooth, ZigBee, IR, RF); each protocol is uniquely optimized to work within a range of operational constraints. While diversity is beneficial because it allows for a high level of tailored performance, it also creates significant challenges to interoperability and standardization. When discussing power supply systems, LiPo batteries are still the most common solution in use, although some innovative ideas are emerging, including dual-battery systems, wireless charging mechanisms, and even some energy-harvesting systems. Power usage and limited runtime are the main challenges when scaling a swarm or deploying swarms. Furthermore, the necessity of adding some flexibility to design practices is highlighted by the inherent trade-off between cost-effectiveness and function. A promising way to achieve extensibility from general-purpose platforms without compromising economies of scale is through modular hardware solutions. The absence of hardware standards continues to be a major obstacle to cross-platform compatibility and large-scale deployments in swarm robotics, despite significant progress in the areas of hardware integration, energy efficiency, and system miniaturization.

## 4. Software Design

A “software is the product that software professionals build and then support over the long term. It encompasses programs that execute within a computer of any size and architecture, content that is presented as the computer programs execute, and descriptive information in both hard copy and virtual forms that encompass virtually any electronic media” [56]. Any software created for SR aims to achieve swarm behavior and make it possible for this emergent property to arise from basic local rules. Regarding the hardware, Nadjah and Junior [14] present SR software as a promising but fragmented field that requires more standardization and guidance in order to successfully transition to real-world applications. The adoption of methods like automatic design and the establishment of shared foundations are emphasized as critical next steps. The software that permits and controls the collective intelligence and coordination of individual robots is at the core of swarm robotics. Swarm Robotic systems’ software components handle tasks and the application of swarm algorithms, which determine the swarm’s emergent behaviors. Table 2 provides a comparative overview of the SR platforms examined in this paper, thereby synthesizing current knowledge and practices in Swarm Robotics software development.

### 4.1. Modular and Framework-Based Architectures

- SwarmUS Platform

É. Villemure et al. [15] present an innovative approach to swarm robotics development by combining both a software and hardware framework that can work with existing robots. This review is limited to the software components of SwarmUS, which includes a number of components: HiveMind is the firmware that runs on the Hiveboard; HiveConnect is the firmware that handles Wi-Fi networking on the ESP32; HiveMindBridge is a C++ library that handles communication with host robots; Buzz Programming Language is used to write swarm behaviors; and HiveAR is an Android-based app for humans to interact with the swarm. There is also the ability to integrate SwarmUS Buzz for the development of swarm behaviors. The framework features abstraction and modularity, which allows flexibility in robot integration by separating Swarm Intelligence in HiveMind from the robot functions in HiveMindBridge. This framework supports simulation and implementation in real-world deployments or experiments. The advantage of being able to cross-compile Buzz scripts into ROS nodes and use the Gazebo simulation also makes it easier to test and develop behaviors before trying the behaviors on real robots.

- ROS-Based Implementations

HeRo [28] is an SR platform that integrates with ROS, offers both modularity and reusability since it utilizes existing ROS packages, provides a standard method of communication between the robot and connected computers, and employs simple Arduino firmware focused on the essentials, sensors, motors, and ROS communication. Madridano et al. [70] outline a software architecture for autonomous UAV swarms developed for firefighting with an architecture that uses ROS. A software architecture with modularity which follows a layered approach (4 layers) allows independent development and testing, and improves scalability and future work. Good path planning was produced with the PRM-based global path planner, producing good paths for each individual UAV, which included types of formations. The path improved with line-of-sight smoothing, which reduces mission duration as well. Both approaches offer centralized and decentralized methods to prevent collision by managing the inter-UAV collision. Deep Reinforcement Learning (DRL) gives UAVs the ability to autonomous and make decisions about how to avoid obstacles while optimizing their paths in new environments. Swarmie project [66] is a Swarm Robotics platform developed at NASA Kennedy Space Center; it comes with strong choice about ROS framework providing modularity, inter-process communications, standard message types, user interface tools, simulation, and it focuses on code re-use and code collaboration. This philosophy of separating functionality into modular nodes is a sound design principle. Gansari and Buiu [68] presented a novel approach to taking heterogeneous swarms of robots through the ROS-based software framework. The proposed system provides a solution to the problem of integrating heterogeneous robots that have varying hardware and software characteristics. This is particularly important for real-world applications where heterogeneous robot capabilities are required. The system integrates a variety of ROS backbones to provide many benefits, modularity (using the ROS nodes), scalability (ROS’s network characteristics make the system scalable to larger swarms), flexibility (The five working modes), and being open source.

### 4.2. Layered and Hierarchical Architectures

- Zooids Interactive Platform

Concerning Zooids [26], the software architecture that permits complex swarm behaviors and user interaction with the robots is highlighted in their paper. A layered architecture is introduced, consisting of an application layer that specifies the desired swarm behaviors and goals, a simulation layer, a server layer that dispatches commands, and a hardware layer. The Zooids are controlled using techniques such as PID control for accurate individual robot positioning, HRVO for real-time collision avoidance, and the Hungarian Algorithm for effective swarm reconfiguration. The full potential of such swarm systems may be unlocked by addressing their limitations and advancing their development.

- Aquatic Systems Architecture

A well-organized and thorough software architecture for managing and controlling a swarm of aquatic robots is described in Costa et al.’s [25] project, Design and Development of an Inexpensive Aquatic Swarm Robotics System. The software includes a Raspberry Controller. It runs on a Raspberry Pi running Raspbian OS on each robot. It has an intuitive interface and makes good use of open-source libraries (Pi4J, WiringPi) for hardware interaction, encouraging code reuse and community participation. In addition to logging commands and messages for offline analysis and debugging, it permits real-time control and deployment of waypoints, geo-fences, and obstacle information. To facilitate communication between robots and between robots and base stations, the system makes use of a Wi-Fi network.

- Fast Low-Cost Systems

Bartmess et al. [33] create a fast, low SR, modular design with distinct vision processing and robot control modules that enhance clarity and maintainability, a UDP communication protocol, and a well-known ORB algorithm and vision targets for localization. Finally, the paper shows a good basis for a low-cost SR system. By addressing the possible issues and investigating other features, the system’s scalability, flexibility, and robustness can be further improved.

### 4.3. Educational and Human–Robot Interaction Platforms

- Cellulo Educational Platform

The Cellulo paper [30] introduces a novel and unique approach to educational robotics, based on the use of a swarm of inexpensive, haptic-enabled, and compact robotic units. The platform’s objectives of adaptability, usefulness, and ubiquity in educational robotics are all successfully supported by the software design, which is essential to reaching these objectives. The adaptable and scalable design holds promise for future research and development of Swarm Robotics in education, despite certain drawbacks. The decentralized architecture includes a number of software components, including a tablet with a user interface, a haptics controller, Bluetooth 2.1, and vision-based technology.

- Safety-Focused Remote Platforms

With an emphasis on simulation-based verification, safety barriers, and user-friendly interfaces, the Robotarium [31] software exhibits a well-designed method of enabling safe (using the Safety Measures through simulation-based verification and safety barrier certificates). The software is a useful tool for developing the field because of its focus on accessibility and safety.

### 4.4. Automatic Design and Control Software Generation

- AutoMoDe Family

Salman et al. [16] provide useful details regarding the automatic development of robot swarm control software, emphasizing financial constraints. They present the AutoMoDe framework’s “Waffle” platform, which focuses on assembling and optimizing pre-defined modules for mission-independent control software. They create control software using Probabilistic Finite State Machines (PFSMs), which dictate robot’s actions based on internal parameters and sensor input. This provides flexibility and less human involvement, enabling mission-specific module combination and selection, and it automatically adjusts each module’s parameters to improve performance for the selected mission. All of this can be done while adhering to financial constraints, which have a big impact on software design and the behaviors that result.

Next we discuss the paper by Francesca et al. [58], which proposed an automatic control design method for swarm robots called AutoMoDe-Chocolate. The paper compares Chocolate to the previously mentioned automatic design methods and manual design methods: The paper starts with a reference model that describes capabilities of the e-puck robot, which includes formalization of sensor’s input and actuator outputs and a standardized setting to allow comparison of results. The first automatic design methods described were Vanilla, with pre-defined modules that create a probabilistic finite state machine and optimizes it using F-Race; EvoStick, which evolves a feedforward neural network with an evolutionary algorithm; and Chocolate, which builds on Vanilla by utilizing an iterative version of F-Race to find better optimal solutions. The author also highlights the manual design methods including U-Human (unrestricted design by a human expert) and C-Human (constrained design using the same modules as Vanilla and Chocolate).

In his doctoral thesis [71] on “the automatic modular design of control software for robot swarms,” Hasselmann addresses higher-level design methodologies, architectures, and bridging simulation–reality gaps. He introduces direct neuroevolution and modular neural network behaviors as Control Software Architectures and mentions behavior trees as an alternative. Hasselmann is a good source of information about SR software.

Returning to the work of Francesca et al. [59], they developed Vanilla in 2014 and then Chocolate in 2015. Chocolate has been referenced previously in this review, but with regard to Vanilla, their paper compared approaches for the design of control software for swarm robots, specifically dealing with the e-puck robot platform. The software architectures that they compared were a follows:

1. AutoMoDe-Vanilla (Vanilla) is a modular approach-based automatic design process. It assembles pre-existing parametric modules that are representations of low-level behaviors and conditions (such as black-floor, neighbor-count, etc.) and synthesizes control software in the form of a probabilistic finite state machine.
2. EvoStick is an implementation of evolutionary robotics for automatic design, and it deals with an unhidden feed-forward neural network.
3. U-Human is a manual design approach, where human experts use the API to the robot’s sensors and actuators in the creation of control software with complete freedom.
4. C-Human is an additional manual design approach, where human experts use the same parametric modules and control architecture as Vanilla.

The project places a strong emphasis on modular design, especially in the Vanilla and C-Human approaches, which use pre-existing modules to limit the plan space. By striking a balance between interpretability and complexity, this methodology aids in closing the reality gap. A probabilistic finite state machine (PFSM) architecture is used by both Vanilla and C-Human, which makes them ideal for simulating robot swarm behavior. EvoStick, on the other hand, uses neural networks, which provide more representational power but run the risk of overfitting, which can widen the reality gap.

Kuckling et al. investigate the use of behavior trees as a control architecture for automatically creating swarm robot software in their paper [60]. It introduces a brand-new technique called Maple that builds and optimizes existing modules into a behavior tree. It presents a novel use of behavior trees in the design of automatic swarm control. It enhances expressiveness with two-way control transfers and sub-trees to enable complex behaviors. It is tested on actual e-puck robots, and the results demonstrated performance that was either on par with or better than alternative approaches.

Another method for automatically creating robot swarms with communication-based behaviors is called Gianduja [72]. The study examines the software design process and uses both simulations and real-world experiments to assess the control software that is produced. Its modular design approach enables reactive and adaptable behavior according to probabilistic rules. The AutoMode family of robots communicates by means of a single locally broadcast message with emergent semantics, which means that the interpretation of the message is dependent on the evolved behavior.

- Machine Learning and Evolutionary Approaches

The diversity of software approaches is clearly highlighted in Francesca and Birattari’s [69] paper, with a shift towards modularity presenting the need for more structured research, comparisons, and benchmarking in the field of automatically designing control software for Swarm Robotics, particularly leveraging evolutionary techniques. They cited parametric control architectures, modular architectures, probabilistic finite state machines, and monolithic neural networks as examples of how establishing standards would allow for the objective evaluation of various software designs and optimization techniques. Furthermore, they discussed online techniques, such as embodied evolution, that allow robots to adapt software while they are in operation, as well as offline techniques that use simulations and evolutionary algorithms.

### 4.5. Specialized Platform Implementations

- Low-Level Programming Approaches

In the paper by Abuelhaija et al. [32], Atmel Studio is used to program the robots in AVR assembly. The code is divided into subroutines. A particular task, such as motor control, sensor reading, or executing a portion of the AI algorithm, is probably handled by each subroutine. Interpreting sensor data and converting it into robot movement is the main function of the AI algorithm. The relationship between IR sensor readings—which indicate distance to other robots—and the amount of time spent moving forward is established by the second-order polynomial equation presented in the paper.

- Large-Scale Self-Assembly Systems

Rubenstein et al.’s paper [57] introduces some algorithms to realise the self-assembly behavior in a thousand SRs called kilobots. All Kilobots run an identical program containing the self-assembly algorithm and target shape image, highlighting the decentralized nature of the system with no central controller. Edge-following, gradient formation, and localization through neighbor trilateration are the three basic collective behaviors they employ; these primitive behaviors are combined into a finite-state automaton that dictates each robot’s sequence of actions based on its current state and sensor data. It is an innovative algorithmic design that enables complex collective self-assembly to result from interacting with numerous basic robots with restricted capabilities; however, there is a lack of details about the used software.

- Specialized Domain Applications

Another SR platform is mROBerTO [40]. Its software, which is programmed in C++ using an open-source SDK for greater control, has an onboard ARM processor that enables sophisticated swarm algorithms and communication via ANT and Bluetooth Low Energy (BLE).

### 4.6. Communication and Standardization Frameworks

- Cross-Platform Communication

Returning to the aforementioned standardization issue, SwarmTalk [62] is a significant step toward benchmarking and standardized cross-platform communication for Swarm Robotics. This paper addresses the critical need for standardized Swarm Robot communication and emphasizes efficiency, portability, and ease of use. It has a portable design with a straightforward driver interface for cross-platform use and minimal resource requirements for memory-constrained platforms.

- Domain-Specific Languages

Next is Buzz [61], which is a programming language oriented towards SR design. Buzz introduces important innovations like the swarm construct for more nuanced, dynamic swarm behaviors. The extensible design, situated communication, and stigmergy mechanisms provide powerful tools for heterogeneous swarm programming.

### 4.7. Development Toolkits and Research Platforms

- Python-Based Development Tools

By emphasizing behavior implementation in a cross-platform, the Python-based package, Pyswarming, a valuable contribution enabling Swarm Robotics research, provides de Andrade et al. [63] with a toolkit that is extremely helpful and makes the work much easier for the developers. Accessibility, an existing algorithm library, ease of use, and adaptability are among its advantages.

- Actor-Oriented and Distributed Systems

In the case of SR, Akkaya et al. [65] present PILOT, a software toolkit intended for developing data-intensive distributed applications in robotic swarm scenarios. PILOT offers a novel actor-oriented paradigm that is ideal for modular swarm programming. Toolkits are very helpful to developers in all CS branches for facilitating. This is especially beneficial due to its machine learning components, ability to handle streaming data, and clear state-space representation. However, there are issues in evaluating its effectiveness as a complete swarm software toolkit without information regarding the algorithm library, implementation, scalability, and the scope of learning integration.

- Platform-Agnostic Solutions

A hardware/software suite that enables swarming behavior in a range of robots is described by Chamanbaz et al. [64]. The software’s ”marabunta,” a Python module, has a number of notable characteristics:

1. Robot control “body,” communication “network,” and swarming behavior “behavior” are all divided into separate classes in a modular design.
2. Platform agnostic: By developing body classes tailored to a particular platform, various robots can be integrated (eBot and e-puck examples provided).
3. Heterogeneity is enabled and communication is flexible.
4. It offers the “MockBody” and “MockNetwork” classes for quick prototyping and simulation of swarm algorithms without the need for actual hardware. Additionally, Python (3.13.7) is used for ease of development.

### 4.8. Formal Methods and Verification Approaches

- Property-Driven Design

”Property-Driven Design” is a novel approach to designing Swarm Robotics software that Brambilla et al. [67] present. It promotes a top-down strategy that focuses on desired properties and formal verification through model checking in place of the conventional “code-and-fix” method. In terms of formal specification and verification, the property-driven design approach offers a promising formal methods-based approach. It is possible to formally specify desired swarm behaviors and validate them through model checking by utilizing PCTL and Markov chains. This lowers the possibility of unforeseen consequences and helps guarantee that the software meets its objectives. Design is given precedence over implementation specifics in property-driven design by advocating for the concept of a prescriptive model that serves as a guide.

### 4.9. Historical Context and Comparative Analysis

- Multi-Robot Systems Overview

A comprehensive review of multi-robot systems with a focus on hardware is given by Parker et al. [73]. The paper mainly emphasized hardware, but it also covered what are considered important software concepts related to swarms: behavior-based control, where simple reactive behaviors reactively combine to create the emergent swarm behaviors; and distributed algorithms for swarm behaviors, like leader following, dispersion, and clustering.

### 4.10. Critical Languages and Techniques

Swarm robotics software relies on a diverse set of programming languages and strategies across multiple levels depending on the design goals and platform. At low-level programming, C and C++ are essential for embedded systems (e.g., mROBerTO, AVR-based robots, etc.), with their direct access to hardware and moderate level of efficiency. Python is growing to be one of the preferred programming languages for simulation, prototyping, and educational platforms, with a fairly simple language and repository of algorithm libraries (e.g., Pyswarming, Marabunta). DSLs (domain-specific programming languages, e.g., Buzz) offer constructs specifically designed for swarm-based programming using principles such as distributed coordination and stigmergic communication. Next, middleware frameworks have become even more prominent to facilitate modularity, interoperability, and real-to-simulation continuity across heterogeneous robots (especially recognized with the Robot Operating System, ROS). Finally, a few relatively simple methods like automatic design (e.g., AutoMoDe, probabilistic finite state machines, behavior trees) and a few machine learning approaches (e.g., genetic programming with neural networks, reinforcement learning, etc.) are now commercially scalable routes for generating swarm behavior with minimal manual programming. With different trade-offs of efficiency, modularity, and adaptability, the options and strategies when developing swarm robotics software are greater than ever before.

### 4.11. Summary of the Software Section

**Figure 12:**
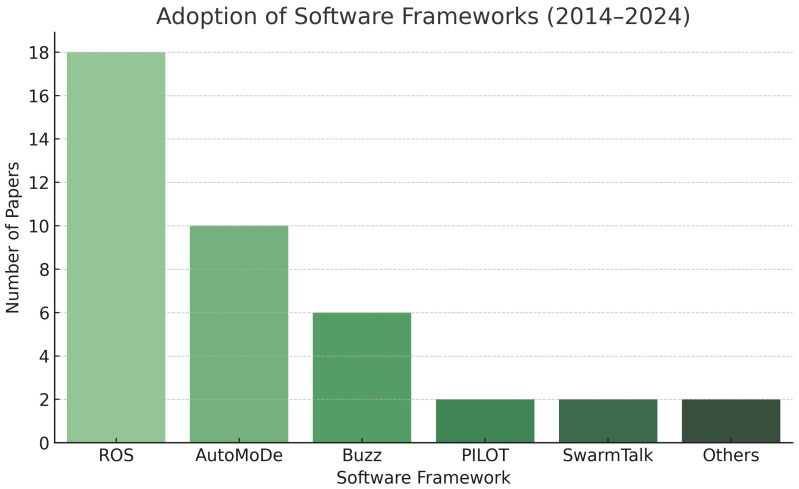
The adoption frequencies of the software frameworks across the 40 papers. ROS is dominant due to its modularity and community involvement; however, the AutoMoDe family has made substantial contributions in creating automated swarm behavior. Newer frameworks, such as Buzz and SwarmTalk, show promise for heterogeneous swarm control and decentralized swarm control.

From 2014 to 2024, there has been an aggregated development in the Swarm Robotics software becoming more modular, scalable, and standards-based. Instead of being platform-specific, the communication between layers in frameworks (largely ROS) is moving toward reusable platforms. There is greater modularity in automated design paradigms (AutoMoDe) and also domain-specific and decentralized languages (Buzz), which have removed some complexity when designing a Swarm Robotics system. There isalso better communication with decentralized coordination, and emergent behavior has moved swarm development and deployment towards ways of integrating simulation-to-reality as a core element of systems design. While the application of machine learning for adaptive control has arguably not been used in Swarm Robotics, there are, in some sense, areas of interest. The issues of standardization or interoperability are touching on established examples like SwarmTalk and middleware abstractions, although systems that stack abstraction on top of each other seem to be getting ahead of the practice of standardization. Overall, there is movement away from or an absence of ad hoc experimental settings towards more familiarized, comfortable, and production-ready software ecosystems. Figure 12 illustrates the adoption frequencies of the software frameworks in SR.

## 5. Answering the Research Questions

Based on the systematic review conducted on the 40 peer-reviewed articles, this section will provide a critical analytical review of four key research questions.

### 5.1. What Are the Key Standardization Barriers That Prevent Swarm Robotics Deployment in Real World Applications?

According to our analysis, Swarm Robotics transition from laboratory to real-world deployments is severely constrained by standardization barriers, which constitute a complicated and interrelated web of methodological, technical, and financial difficulties.

1. Hardware Fragmentation and Platform Incompatibility:
  The lack of unified hardware architectures is the most significant obstacle. More than 20 different hardware platforms were found during our review; these platforms used various sensor configurations, communication protocols, and power management systems. For example, SwarmUS [15] incorporates RPLidar and Realsense cameras, the Colias robot [27] uses only infrared proximity sensors, and aquatic platforms such as Jeff [29] use electrodes and pressure sensors for underwater navigation. This diversity creates several compounding problems:
  - The inability to migrate across platforms: Research findings are not transferable because each platform necessitates extensive software rewrites.
  - Fragmentation of the component supply chain: Specialized Swarm Robotics components do not have economies of scale.
  - Inconsistencies in testing and validation: Performance comparisons across platforms are useless due to disparate sensor capabilities.
2. Communication Protocol Chaos:
  Possibly the biggest obstacle to standardization is the communication environment. Among the platforms we examined, our analysis found at least seven distinct communication methods: Bluetooth, ZigBee, infrared, RF modules, electric field detection, Wi-Fi (IEEE 802.11), and even acoustic signals. This disarray shows up in a number of important ways:
  - Interoperability impossibility: Heterogeneous swarms are prevented by the inability of robots from different platforms to communicate.
  - Mismatches in range and bandwidth: ZigBee systems (800–1000 m range) cannot be coordinated with robots that have Bluetooth (10–15 m range).
  - Changes in protocol overhead: The latency and power consumption of various communication stacks vary greatly.
3. Software Architecture Divergence:
  Equally problematic is the software standardization barrier. Our analysis revealed essentially distinct architectural methodologies:
  - Monolithic systems that closely integrate swarm logic and hardware control (such as Costa et al.’s aquatic SR [25]).
  - Layered architectures that contain layers for applications, simulations, servers, and hardware (like Zooids [26]).
  - Modular frameworks that isolate Swarm Intelligence from robot functions, such as SwarmUS [15].
  - Automatic design systems (AutoMoDe variants [58,59,60]) that use algorithms to create control software.
  Because each approach necessitates distinct programming paradigms, development tools, and deployment methodologies, these architectural differences create unsolvable integration challenges.
4. Economic Constraint Modeling Gaps:
  The absence of standardized economic modeling for swarm systems presents a particularly pernicious standardization obstacle. Salman et al. [16] were the only ones to specifically address economic constraints in their Waffle platform, demonstrating how cost constraints radically alter software design and hardware selection. Standardized cost–performance metrics are lacking, which prohibits the following:
  - Selecting a platform rationally: Developers are unable to compare platforms for particular applications in an unbiased manner.
  - Planning an investment: Businesses are unable to forecast maintenance needs or scaling expenses.
  - Evaluation of risk: Swarm deployment’s economic feasibility is still mostly up in the air.
5. Testing and Validation Methodology Inconsistencies:
  Standardized testing procedures are critically lacking, according to our analysis. The only attempt at a standardized experimental infrastructure is the Robotarium [31], but even this platform has hardware limitations that prevent wider use. This results in the following:
  - Reproducibility issues: It is impossible to confirm experimental findings on various platforms.
  - Inconsistencies in performance metrics: The success metrics used in different studies differ significantly.
  - Challenges with safety certification: There are no established methods for assessing swarm safety by regulatory agencies.

### 5.2. How Do Hardware/Software Integration Choices Affect Swarm Performance and Scalability?

According to our analysis, the choices made regarding hardware–software integration have a cascading effect that essentially determines swarm capabilities. While some architectural choices allow for scalability, others place strict restrictions on system performance.

- Sensing–Communication–Control Integration Patterns

The most effective swarm implementations show close coordination between control algorithms, communication protocols, and sensing capabilities. Our analysis reveals three unique integration patterns:

*Pattern 1: High Scalability Minimalist IntegrationPlatforms such as Kilobots [57] use extreme hardware–software minimalism to achieve remarkable scalability (1000+ robots). The following are part of the integration strategy:

1. Complex control algorithms are eliminated by using simple vibration motors for locomotion.
2. Protocol overhead is almost eliminated with IR communication.
3. Coordination complexity is eliminated when all agents have the same programming.

This integration choice enables massive scalability but severely limits behavioral complexity and environmental adaptability.

*Pattern 2: Balanced Performance through Multi-modal IntegrationMultiple sensor types (ultrasonic, infrared, and cameras) and adaptable communication options (Wi-Fi, Bluetooth, and ZigBee) are integrated by platforms such as the UBswarm [24]. This results in the following:

1. Improved environmental awareness by fusing sensors.
2. The choice of communication protocol according to mission requirements.
3. Software architectures that are modular and capable of utilizing various hardware capabilities.

But due to the high complexity costs associated with this flexibility, practical swarm sizes are limited to dozens of agents instead of hundreds.

*Pattern 3: Specialized Integration (Low Scalability, High Performance) Highly specialized integration is used by sophisticated platforms such as Jeff [29] to achieve sophisticated behaviors:

1. Multimodal sensing (electrodes, magnetometry, pressure, and acceleration).
2. A variety of communication channels, including electric field, acoustic, and LED.
3. Advanced autonomy algorithms for navigation under water.

Due to financial and computational limitations, this integration approach significantly restricts scalability even though it permits complex behaviors.

- Power Management and Performance Trade-offs

Performance and scalability are at odds with three integration strategies:

1. Optimal power allocation, extended operation (up to 120 min for Jeff [29]), and support for demanding sensors are all made possible by centralized power management (e.g., SwarmUS [15]); however, scalability is limited due to increased cost and complexity.
2. Though it limits behavioral sophistication by reducing sensor/processing capacity, distributed power management (e.g., Cellulo [30]) lowers cost and complexity, allowing for larger deployments and simpler maintenance.
3. Although they increase per-agent costs, hybrid systems (e.g., Abuelhaija et al. [32]) improve accuracy, subsystem optimization, and fault tolerance by separating power for motors and control circuits.

- Communication Architecture Scalability Limits

Scalability is significantly influenced by communication integration:

1. Large swarms are fragmented by broadcast systems (common in IR-based designs) due to quadratic message collisions, bandwidth saturation, and range constraints.
2. Although network-based systems (like the Wi-Fi in Robotarium [31]) offer infrastructure support, higher bandwidth, and structured routing, they also limit deployment flexibility.
3. Long-range networking (ANT, BLE) and short-range sensing (IR) are combined in hybrid communication (e.g., mROBerTO [40]), which optimizes coordination at multiple scales at the expense of high software complexity.

- Control Algorithm–Hardware Coupling Effects

Scalability and performance are determined by the degree of algorithm–hardware coupling:

1. Although it is brittle and non-transferable, tight coupling (such as aquatic control in Jeff) maximizes efficiency and predictability in particular environments.
2. Portability, modularity, and maintainability are made possible by loose coupling (such as hardware abstraction in SwarmUS ), albeit at the expense of performance and limited hardware exploitation.

### 5.3. Which Design Patterns Show up in Swarm Robotics Implementations That Are Successful Versus Those That Are Unsuccessful?

Our comparative review of the 40 papers identifies unique architectural and methodological trends that are highly related to implementation success as determined by adoption by the research community, scalability accomplishments, and real-world deployment viability.

- Successful Implementation

As demonstrated by AutoMoDe-based systems [58,59,60] and Kilobots [57], the first is the idea of emergent complexity through radical simplification. These platforms eliminate coordination overhead and instead take advantage of emergent collective dynamics by purposefully limiting individual agent capability to the bare minimum—using only vibration motors and infrared communication while running identical software. Unprecedented scalability is made possible by this drastic simplification; multiple research teams have demonstrated that more than 1000 coordinated robots are capable of collective transport and self-assembly. The effectiveness of this strategy highlights a crucial realization: scalability arises when coordination complexity is decreased as opposed to when agent capabilities are increased.

Platforms like SwarmUS [15] and the Robotarium [31]) exhibit the second successful pattern, which is modular architectures with clearly defined abstraction boundaries. Such systems manage complexity within modularity through clean separation of Swarm Intelligence layers (e.g., HiveMind) and hardware-specific functionality (e.g., HiveMindBridge). This provides independent development, cross-platform portability, and smooth transitions from simulation to deployment. The primary scaling component over time will be modularity, because it provides continuous improvement without requiring new designs, as was shown by their adoption by a range of research teams and validated efficacy in conditions outside of laboratory environments. Standard interfaces and area-specific optimization are the fundamental elements of the third example, especially obvious in specialized areas such as aquatic systems (Costa et al. [25]) and aerial swarms (pico quadrotors [36]). By following standardized communication protocols, these platforms achieve this compatibility while allowing for a hardware and algorithmic customization within defined environmental constraints. They achieve robust domain performance, successful deployment in the field, and even commercial translation only by coordinating actual operational considerations with optimization. This balance between specialization and interoperability allows Swarm Robotics to take steps toward practical applications.

- Unsuccessful Implementation

Failed implementations of Swarm Robotics will typically have some combination of single points of failure, hardware–software mismatches, and over-generalization, which leads to failed performance, poor scalability, and inefficiency. Although there are no direct SPOFs identified in any of the reviewed 40 papers, the increased use of case studies in the broader swarm robotics literature does show instances of how systemic SPOFs exist. For instance, the Robotarium testbed introduces a central SPOF, as the entire swarm is dependent on its central server to facilitate swarm tracking; if the server and tracking capability fail, the experiment ceases [31]. Similarly, base-station architectures relied upon for charging a swarm or to assign tasks create SPOF dependencies, where if the base-station fails, the entire swarm fails with it. Additionally, in software, dependency on homogeneous swarms executing the same control code allows for logical SPOFs: a single bug in navigation or collision avoidance will propagate through all robots, causing them to all fail to navigate clearly [58,59,61,63]. A Kilobot platform example illustrates a hardware–software mismatch, where the imprecision of vibration-based locomotion was extremely surface dependent, which invalidated behaviors designed in idealized simulations [34,43,57]. Environmental dependencies can also create SPOFs: if a swarm relies on fiducial markers, or ceiling lights for localization, and if those markers or lights are obscured or changed, then the entire swarm fails in those examples [25,29,42]. These examples show that systemic SPOFs exist that are due to limitations not only in hardware, but due to centralized infrastructure, logic in software, and unrealistic assumptions made regarding the environment in which a swarm functions.

## 6. Future Research Directions

Of the many possible research domains in swarm robotics, energy efficiency and standardization are the most critical and fundamental priorities. Energy constraints have always limited the autonomy of swarm systems, since low-cost robots typically have small batteries with limited capacity. Thus, there is a need to improve the underlying hardware for low-power consumption, establish energy-aware algorithms, and explore alternatives such as wireless charging docks and energy-harvesting techniques. Until important progress is made in these areas, the feasibility of large-scale, long-term duration swarm deployments would be continually out of reach. Another compelling issue surrounds the lack of standardized interfaces and communication protocols that would allow more interoperability across platforms that have different characteristics. Different robots use variations of communication frameworks (Bluetooth, ZigBee, RF, Wi-Fi, infrared, etc.), which prevents scaling up for collaborative tasks in an efficient manner. Rather, combinations of adaptive and hybrid communication, partnered with an agreed-upon standard, could realize much more compatibility across platforms and flexibility for integration. Once these two fundamental areas have been addressed, the foundation of improvements, such as the use of machine learning methods, new application domains, and theoretical models, could be implemented in much better ways. Only once advances across these two barriers are made will Swarm Robotics be able to advance from experimental testbeds toward real-world application.

1. Standardization and Interoperability
  - Hardware Standardization: A universal hardware interface and reference architectures would allow interoperability across different platforms. Many of the studies included in the review used incompatible hardware, resulting in over 20 different hardware platforms and creating significant deployment barriers.
  - Communications Standardization: Adaptive communication protocols that allow swarms to switch between the identified communication methods (seven in total: Wi-Fi, Bluetooth, ZigBee, IR, RF, acoustic, electric field) as mission requirement dictators is required.
2. Machine Learning Incorporation
  - Distributed Learning: It is integral that federated learning algorithms are developed for swarms to adjust behaviors on-the-fly while maintaining a decentralized swarm.
  - Emergent Behavior Predictability and Control: Before effective emergent behaviors can even be considered for application, the development of a mathematical framework for predicting emergent behaviors with individual robot programs will be necessary.
3. Scalability
  - Ultra-Large Scale Architecture: With Kilobots demonstrating how 1000 or more robots can operate within a swarm, protocols and hierarchical control structures governing communication at these massive scales must be developed.
  - Energy-Efficient Operation: Swarms will need to be able to operate indefinitely using the various wireless charging networks and multi-modal energy harvesting to extend their current 40–120 min of operations.
4. Application-Oriented Research
  - Environmental Monitoring: Investigate development of sustainability-friendly, multi-modal sensing systems comprised of aerial, terrestrial, and aquatics robots to better understand ecosystems.
  - Disaster Response: Develop swarms of robots that work collaboratively, whilst being safe in hazardous environments, and in real-time with human operators.
  - Industrial Application: Research applications for manufacturing and infrastructure inspection, specifically with bundled, swarm-like coordination.
5. Theoretical Development
  - Formal Verification: Develop model checking and safety certification for swarm systems so they can be deployed in safety-critical applications.
  - Economic Modeling: Develop comprehensive cost-performance models aligned with the economic limitations that influence how systems are designed.
6. Critical Priorities
  - The most significant priority area is standardization—without some basic standardization in the form of common frameworks, we will make no tangible progress, nor will we have commercially viable applications. At the same time, infusing adaptive intelligence via machine learning can create a form of swarm capability that is important for a swarm of robots to operate in a real-world context.

## 7. Conclusions

In summary, this systematic literature review examined publications from the last decade regarding advancements in the Swarm Robotics field focusing on hardware–software interplay. Following a rigorous process, this review presents a wealth of information on both hardware and software aspects. The review highlights trending technologies in hardware (sensors, networking, actuators, power supply) and software (HAL, ROS, individual robot control, SI algorithms, simulation tools, communication protocols). These aspects are not limited to swarm robotics, but they have more general applicability across the field of robotics. Infrared and ultrasonic sensors will continue to be of foremost importance to navigation and obstacle avoidance in mobile robots and service robots; vision-based sensing will remain useful in tasks like localization, mapping, and manipulation. More sophisticated examples, like impedance and admittance control, illustrate how sensor feedback can be leveraged during human–robot interaction, particularly when physical contact occurs. For example, the techniques examined in this review could also be applied to [74] where reliable sensing is critical for ensuring safe and adaptive collaboration. Moreover, it discusses the trends in integrating machine learning and modular architectures into the field. The review also identifies critical gaps in the field, including the urgent need for standardization of the hardware and the software of SR. It also examines economic constraints and their impact, proposes solutions, and addresses environmental diversity challenges. The paper covered the opportunities for future research in the field.

Finally, the review offers a concise overview of trending technologies from the last decade that can help researchers by providing insights to laying groundwork for advancing the swarm robotics field, emphasizing the importance of interdisciplinary collaboration and innovation to unlock the full potential of these systems in both academic and practical applications.

## Figures and Tables

**Table 1:** Summary of hardware components used in Swarm Robotics platforms.

Category	Component	Function/Usage	Specs (Power, Range, Cost)	Platform(s)/Ref.
**Sensors**	GP2Y0A21YK IR Sensor	Obstacle avoidance; range detection	30–50 mA; 10–80 cm; $5–10	S-Bot [23]
	HC-SR04 Ultrasonic	Distance measurement; anti-collision	15 mA; 2–400 cm; $1–5	UBswarm [24]; Mustafa SR [23]
	Realsense D400 Camera	Depth sensing; 3D mapping	1.6 W; USB; $150–300	SwarmUS [15]
	DS18B20 Temp Sensor	Water temperature sensing	1 mA; digital; $2–10	Aquatic SR (Costa) [25]
	AT42QT1070 Touch Sensor	Capacitive human interaction	2 µA; $5–15	Zooids [26]
	LSM303D Magnetometer	Orientation and heading	100 µA; $5–20	Aquatic SR (Costa) [25]
**Actuators**	DC Gear Motors (Solarbotics)	Wheeled movement	35–150 mA; 3–12 V DC; $10–30	Colias [27]; UBswarm [24]; S-Bot [23]
	SG90 Servo Motors	Wheel drive (modified)	250 mA peak; 5 V; $2–10	HeRo [28]
	Piezoelectric Actuators	Micro-scale movement	mW range; low voltage; $10–50	Kilobot [29]
	Omni-wheel Drive	Holonomic motion	Variable; platform-specific $10–50	Cellulo [30]
	Propeller Motors (NTM, EMAX)	Aquatic propulsion	140 W; 11.1 V LiPo; $10–30	Jeff [29]; Costa SR [25]
	Stepper Motors	Precision wheel control	Platform dependent $10–40	Robotarium [28] (GRITSbot)
**Communication**	ESP8266 Wi-Fi	Long-range communications; ROS link	70–170 mA; 20–50 m; $2–10	HeRo [28]; GRITSbot [31]
	PmodWiFi (SPI)	High-speed Wi-Fi data	250 mA; 400 m; $20–30	UBswarm [24]
	nRF24L01+ RF Chip	RF link with PC/server	15 mA; 800–1000 m; $1–5	Zooids [26]
	RN-42 Bluetooth	App/tablet communication	15–50 mA; 10–15 m; $10–30	Cellulo [30]
	IR Sensors (long/short)	Obstacle avoidance; short-range comms	30 mA; 1–5 m; $5–20	Colias [27]; S-Bot [23]
	TL-WN722N Wi-Fi Adapter	Aquatic robot-to-base communications	200–300 mA; 40 m; $10–30	Aquatic SR (Costa) [25]
**Power Sources**	3.7 V Li-Po Battery	Main control and drive power	600–1200 mAh; USB; $5–15	Colias [27]; Zooids [26]
	11.1 V Li-Po Pack	High power for aquatic missions	880 mAh ×8; 120 min; $20–50	Jeff [29]
	Wireless Charging Dock	Automatic recharge	400 mAh; 40 min runtime; $10–30	Robotarium (GRITSbot) [28]
	Dual Battery (Motor+Logic)	Noise isolation; redundancy	4.2 V Li-Ion ×2; $20–50	Abuelhaija SR [32]
	USB-rechargeable Li-Ion	Educational use; safe	Rechargeable via USB; $5–10	Cellulo [30]

**Table 2:** Analytical comparison of Swarm Robotics software platforms and architectures.

Platform/Architecture	Features	Limitations	Applications
SwarmUS [15]	ROS + Buzz integration, modular, real-to-simulation continuity	Requires experience with multiple tools	Advanced real-world hybrid deployments
Aquatic SR [25]	Real-time Java control, low-cost Raspberry Pi, user-friendly interface	Limited modularity and scalability	Low-cost aquatic swarms
Zooids [26]	Human-swarm interaction, high-frequency coordination, layered architecture	Limited to tabletop	UI/HCI studies
Cellulo [30]	Haptic feedback, education-focused, decentralized design	Limited complexity	Educational robotics
Robotarium [31]	Safety mechanisms, remote testing, real-to-sim pipeline	Limited hardware control	Cloud-based experiments
HeRo [28]	Simple Arduino+ROS setup, cost-effective, modular firmware	Minimal swarm-specific middleware	Teaching platforms
Waffle (AutoMoDe) [16]	Modular, automatically optimized control under constraints	Not flexible beyond mission-specific designs	Automated, constraint-aware controller generation
Kilobots [57]	Extremely scalable, low-cost, decentralized	Lacks computation diversity	Self-organization studies
AutoMoDe-Chocolate [58]	PFSM + optimization with structured modules	Limited expressiveness	Automated control design
AutoMoDe-Vanilla [59]	Simple PFSM composition	Less optimal than Chocolate	Baseline automated design
AutoMoDe-Maple [60]	Modular behavior trees, automatic tuning	Higher complexity	Rich control strategies
Buzz [61]	Swarm-specific DSL, stigmergy	Steep learning curve	Heterogeneous swarms
SwarmTalk [62]	Lightweight communication API	Middleware only	Communication layer
Pyswarming [63]	Pythonic interface, built-in algorithms	Limited to simulation	Teaching, prototyping
Marabunta [64]	Modular Python framework, supports heterogeneity	Requires manual adaptation	Platform-agnostic simulation
PILOT [65]	Actor-oriented toolkit for ML and distributed control	Limited real-world validation	ML-integrated distributed programming
Swarmie [66]	Modular ROS architecture, formation control	Application-specific	Engineering missions
Property-Driven Design [67]	Formal methods; uses PCTL and model checking	High complexity	Safety-critical verification
ROS-Heterogeneous Framework [68]	Five-mode abstraction, modular, ROS-based	Custom-built; limited general documentation	Unified control of heterogeneous multirobot team
EvoStick (Neural Net) [59]	Evolves FFNNs using evolutionary algorithms	Prone to overfitting; lacks interpretability	Optimizing reactive swarm behavior from scratch
Monolithic Neural Nets [69]	End-to-end design; learns complex behaviors	Poor transparency, hard to debug or reuse	Experimental end-to-end control in simulation
Behavior Trees [60]	Modular, hierarchical control flow; easy debugging	Still emerging in swarm robotics context	Tree-based swarm control logic design

## Data Availability

Not applicable.

## Supplementary Materials

The following supporting information can be downloaded at: https://www.mdpi.com/article/10.3390/s25196115/s1, PRISMA 2020 Checklist [13].
