# Supplementary material for: From Network Sensors to Intelligent Systems: A Decade-Long Review of Swarm Robotics Technologies
Source: Sensors (Basel). 2025 Oct 3;25(19):6115. doi: 10.3390/s25196115 (PMC12526905; doi:10.3390/s25196115)
Supplement: Supplementary file 1 [file sensors-25-06115-s001.zip › sensors-3871934-supplementary.pdf]

# PRISMA 2020 Checklist

| Section and Topic | Item # | Checklist item | Location where item is reported |
|---|---|---|---|
| **TITLE** | | | |
| Title | 1 | Identify the report as a systematic review. | Title page (“From network sensors to intelligent systems: A decade-long review of swarm robotics technologies”) |
| **ABSTRACT** | | | |
| Abstract | 2 | See the PRISMA 2020 for Abstracts checklist. | Abstract section (lines 1–20) |
| **INTRODUCTION** | | | |
| Rationale | 3 | Describe the rationale for the review in the context of existing knowledge. | Section 1. Introduction (lines 23–68) |
| Objectives | 4 | Provide an explicit statement of the objective(s) or question(s) the review addresses. | Section 2.1. Research Questions (lines 84–93) |
| **METHODS** | | | |
| Eligibility criteria | 5 | Specify the inclusion and exclusion criteria for the review and how studies were grouped for the syntheses. | Section 2.2. Exclusion & Inclusion Criteria (lines 94–103) |
| Information sources | 6 | Specify all databases, registers, websites, organisations, reference lists and other sources searched or consulted to identify studies. Specify the date when each source was last searched or consulted. | Section 2.4. Execution (lines 128–132) (Searches conducted up to June 2024) |
| Search strategy | 7 | Present the full search strategies for all databases, registers and websites, including any filters and limits used. | Section 2.4. Execution (lines 128–132) |
| Selection process | 8 | Specify the methods used to decide whether a study met the inclusion criteria of the review, including how many reviewers screened each record and each report retrieved, whether they worked independently, and if applicable, details of automation tools used in the process. | Section 2.4. Execution + Fig. 4 (lines 133–139) (One reviewer screened and selected studies (no automation |

| | | | |
|---|---|---|---|
| | | | tools used)) |
| Data collection process | 9 | Specify the methods used to collect data from reports, including how many reviewers collected data from each report, whether they worked independently, any processes for obtaining or confirming data from study investigators, and if applicable, details of automation tools used in the process. | Section 2.4. Execution (data extraction) and Section 2.3 (Quality Assessment Criteria) |
| Data items | 10a | List and define all outcomes for which data were sought. Specify whether all results that were compatible with each outcome domain in each study were sought (e.g. for all measures, time points, analyses), and if not, the methods used to decide which results to collect. | Sections 3 & 4 (Hardware Design, Software Design) + Tables 1–2 |
| | 10b | List and define all other variables for which data were sought (e.g. participant and intervention characteristics, funding sources). Describe any assumptions made about any missing or unclear information. | Sections 3 & 4 (Hardware Design, Software Design) + Tables 1–2 |
| Study risk of bias assessment | 11 | Specify the methods used to assess risk of bias in the included studies, including details of the tool(s) used, how many reviewers assessed each study and whether they worked independently, and if applicable, details of automation tools used in the process. | Section 2.3. Quality Assessment Criteria — used as a risk of bias tool to assess methodological soundness. |
| Effect measures | 12 | Specify for each outcome the effect measure(s) (e.g. risk ratio, mean difference) used in the synthesis or presentation of results. | Not applicable — no quantitative effect measures; narrative synthesis only. |
| Synthesis methods | 13a | Describe the processes used to decide which studies were eligible for each synthesis (e.g. tabulating the study intervention characteristics and comparing against the planned groups for each synthesis (item #5)). | Eligibility for synthesis described in Section 2.4; grouping into hardware/software. |
| | 13b | Describe any methods required to prepare the data for presentation or synthesis, such as handling of missing summary statistics, or data conversions. | Section 2.4. Execution + Sections 3–4 (narrative synthesis of |

| | | | |
|---|---|---|---|
| | | | hardware/software) |
| | 13c | Describe any methods used to tabulate or visually display results of individual studies and syntheses. | Results tabulated in Tables 1–2 and summarized in text. |
| | 13d | Describe any methods used to synthesize results and provide a rationale for the choice(s). If meta-analysis was performed, describe the model(s), method(s) to identify the presence and extent of statistical heterogeneity, and software package(s) used. | Narrative synthesis approach justified in Section 2.4. |
| | 13e | Describe any methods used to explore possible causes of heterogeneity among study results (e.g. subgroup analysis, meta-regression). | Section 2.4. Execution + Sections 3–4 (narrative synthesis of hardware/software) |
| | 13f | Describe any sensitivity analyses conducted to assess robustness of the synthesized results. | Section 2.4. Execution + Sections 3–4 (narrative synthesis of hardware/software) |
| Reporting bias assessment | 14 | Describe any methods used to assess risk of bias due to missing results in a synthesis (arising from reporting biases). | Not assessed (limitation noted in Section 6) |
| Certainty assessment | 15 | Describe any methods used to assess certainty (or confidence) in the body of evidence for an outcome. | N/A |
| **Section and Topic** | **Item #** | **Checklist item** | **Location where item is reported** |
| **RESULTS** | | | |
| Study selection | 16a | Describe the results of the search and selection process, from the number of records identified in the search to the number of studies included in the review, ideally using a flow diagram. | Section 2.4. Execution + Fig. 4 (PRISMA-style flow diagram) "Excluded studies were those not meeting eligibility (duplicates, inaccessible, <4 pages, non-English)" |
| | 16b | Cite studies that might appear to meet the inclusion criteria, but which were excluded, and explain why they were excluded. | Section 2.4. |

| | | | |
|---|---|---|---|
| | | | Execution + Fig. 4 (PRISMA-style flow diagram) |
| Study characteristics | 17 | Cite each included study and present its characteristics. | Section 3 (Hardware Design), Section 4 (Software Design), Tables 1–2 |
| Risk of bias in studies | 18 | Present assessments of risk of bias for each included study. | Section 2.3. Quality Assessment Criteria (lines 104–127) |
| Results of individual studies | 19 | For all outcomes, present, for each study: (a) summary statistics for each group (where appropriate) and (b) an effect estimate and its precision (e.g. confidence/credible interval), ideally using structured tables or plots. | Sections 3 & 4 (summaries of each SR project, with references) |
| Results of syntheses | 20a | For each synthesis, briefly summarise the characteristics and risk of bias among contributing studies. | Section 5 (Synthesis of Trends & Challenges, lines 74–77) |
| | 20b | Present results of all statistical syntheses conducted. If meta-analysis was done, present for each the summary estimate and its precision (e.g. confidence/credible interval) and measures of statistical heterogeneity. If comparing groups, describe the direction of the effect. | Section 5 (Synthesis of Trends & Challenges, lines 74–77) |
| | 20c | Present results of all investigations of possible causes of heterogeneity among study results. | Section 5 (Synthesis of Trends & Challenges, lines 74–77) |
| | 20d | Present results of all sensitivity analyses conducted to assess the robustness of the synthesized results. | Section 5 (Synthesis of Trends & Challenges, lines 74–77) |
| Reporting biases | 21 | Present assessments of risk of bias due to missing results (arising from reporting biases) for each synthesis assessed. | Not formally assessed; noted in Section 6 as a limitation |
| Certainty of evidence | 22 | Present assessments of certainty (or confidence) in the body of evidence for each outcome assessed. | Not applicable — this review is descriptive and does not perform meta-analysis |
| **DISCUSSION** | | | |
| Discussion | 23a | Provide a general interpretation of the results in the context of other evidence. | Section 5 (Discussion of Trends & Challenges) |

| | | | |
|---|---|---|---|
| | 23b | Discuss any limitations of the evidence included in the review. | Section 6: Limitations of the Review (databases restricted to Google Scholar, Semantic Scholar, and Google; English-only; no registration). |
| | 23c | Discuss any limitations of the review processes used. | Section 6: Limitations of the Review (databases restricted to Google Scholar, Semantic Scholar, and Google; English-only; no registration). |
| | 23d | Discuss implications of the results for practice, policy, and future research. | Section 7 (Conclusion, lines 76–78) |
| **OTHER INFORMATION** | | | |
| Registration and protocol | 24a | Provide registration information for the review, including register name and registration number, or state that the review was not registered. | Not registered |
| | 24b | Indicate where the review protocol can be accessed, or state that a protocol was not prepared. | Section 2 (The SLR Protocol) |
| | 24c | Describe and explain any amendments to information provided at registration or in the protocol. | Section 2 (The SLR Protocol) |
| Support | 25 | Describe sources of financial or non-financial support for the review, and the role of the funders or sponsors in the review. | acknowledgments section |
| Competing interests | 26 | Declare any competing interests of review authors. | Standard MDPI declaration at the end |
| Availability of data, code and other materials | 27 | Report which of the following are publicly available and where they can be found: template data collection forms; data extracted from included studies; data used for all analyses; analytic code; any other materials used in the review. | Dataset of 40 included studies available from authors upon request. |

*From:* Page MJ, McKenzie JE, Bossuyt PM, Boutron I, Hoffmann TC, Mulrow CD, et al. The PRISMA 2020 statement: an updated guideline for reporting systematic reviews. BMJ 2021;372:n71. doi: 10.1136/bmj.n71. This work is licensed under CC BY 4.0. To view a copy of this license, visit https://creativecommons.org/licenses/by/4.0/
